# Long-term survival and cause-specific mortality of patients newly diagnosed with tuberculosis in São Paulo state, Brazil, 2010–15: a population-based, longitudinal study

**DOI:** 10.1016/S1473-3099(19)30518-3

**Published:** 2020-01

**Authors:** Otavio T Ranzani, Laura C Rodrigues, Sidney Bombarda, Cátia M Minto, Eliseu A Waldman, Carlos R R Carvalho

**Affiliations:** a Pulmonary Division, Heart Institute (InCor), Hospital das Clinicas (HCFMUSP), Faculdade de Medicina da Universidade de São Paulo, São Paulo, Brazil; b London School of Hygiene & Tropical Medicine, London, UK; c São Paulo Tuberculosis Program, Epidemiological Surveillance Centre “Prof Alexandre Vranjac”, São Paulo Secretary of Health, São Paulo, Brazil; d Centro de Informações em Vigilância em Saúde (CIVS), Coordenadoria de Controle de Doenças, São Paulo Secretary of Health, São Paulo, Brazil; e Department of Epidemiology, Faculty of Public Health, University of São Paulo, São Paulo, Brazil

## Abstract

**Background:**

Long-term survival and cause-specific mortality of patients who start tuberculosis treatment is rarely described. We aimed to assess the long-term survival of these patients and evaluate the association between vulnerable conditions (social, health behaviours, and comorbidities) and cause-specific mortality in a country with a high burden of tuberculosis.

**Methods:**

In this population-based, longitudinal study in São Paulo state, Brazil, we described the 5-year survival of patients who were newly diagnosed with tuberculosis in 2010. We included patients with newly-diagnosed tuberculosis, aged 15 years or older, and notified to the São Paulo State Tuberculosis Program in 2010. We excluded patients whose diagnosis had changed during follow-up (ie, they did not have tuberculosis) and patients who had multidrug-resistant (MDR) tuberculosis. We selected our population with tuberculosis from the dedicated electronic system TBweb. Our primary objective was to estimate the excess mortality over 5 years and within the group who survived the first year, compared with the general São Paulo state population. We also estimated the association between social vulnerability (imprisonment and homelessness), health behaviours (alcohol and drug use), and comorbidities (diabetes and mental disorders) with all-cause and cause-specific mortality. We used the competing risk analysis framework, estimating cause-specific hazard ratios (HRs) adjusted for potential confounding factors.

**Findings:**

In 2010, there were 19 252 notifications of tuberculosis cases. We excluded 550 cases as patients were younger than 15 years, 556 cases that were not tuberculosis, 2597 retreatments, and 48 cases of MDR tuberculosis, resulting in a final cohort of 15 501 patients with tuberculosis. Over a period of 5 years from tuberculosis diagnosis, 2660 (17%) of 15 501 patients died. Compared with the source population, matched by age, sex, and calendar year, the standardised mortality ratio was 6·47 (95% CI 6·22–6·73) over 5 years and 3·93 (3·71–4·17) among those who survived the first year. 1197 (45%) of 2660 deaths were due to infection. Homelessness and alcohol and drug use were associated with death from infection (adjusted cause-specific HR 1·60, 95% CI 1·39–1·85), cardiovascular (1·43, 1·06–1·95), and external or ill-defined causes of death (1·80, 1·37–2·36). Diabetes was associated with deaths from cardiovascular causes (1·70, 1·23–2·35).

**Interpretation:**

Patients newly diagnosed with tuberculosis were at a higher risk of death than were the source population, even after tuberculosis treatment. Post-tuberculosis sequelae and vulnerability are associated with excess mortality and must be addressed to mitigate the tuberculosis burden worldwide.

**Funding:**

Wellcome Trust.

## Introduction

Tuberculosis is an ancient disease that still causes a considerable number of deaths worldwide, and is among the leading preventable causes of death yet to be eliminated.^1^ Vulnerability—when individuals are susceptible to physical, biological, psychological, and socioeconomic stressors and have few resources to cope with these^2^—is the main determinant of tuberculosis epidemics and treatment outcomes^3^ and is considered a fundamental target that must be addressed to mitigate the tuberculosis burden worldwide.^1^, ^4^

Most studies have described the survival of patients with tuberculosis while they received treatment or shortly after treatment completion.^3^ To our knowledge, there are few studies concerning long-term tuberculosis survival (ie, 5 years after diagnosis),^5^, ^6^, ^7^, ^8^ and this knowledge is even scarcer in low-income and middle-income countries.^3^, ^9^, ^10^, ^11^ Moreover, detailed evaluation of causes of death in cohorts with tuberculosis has not received much attention^12^ and there is a paucity of population-based studies that are not focused on tuberculosis and HIV coinfection. Evaluation of survival after tuberculosis treatment and associated causes of death allows quantification of the post-tuberculosis mortality burden and can be useful for planning preventive measures to mitigate post-tuberculosis sequelae.^11^, ^13^

We aimed to describe 5-year survival and causes of death of patients with tuberculosis in a population-based longitudinal study in São Paulo state, Brazil. We hypothesised that patients with tuberculosis would have lower survival compared with the source population, and that vulnerable conditions would be associated with this excess mortality and cause-specific mortality.

**Research in context**

**Evidence before this study**

We searched PubMed for studies that assessed long-term cause-specific mortality for tuberculosis in adults and were published in English, Spanish, or Portuguese, from PubMed's inception until June 30, 2018, with the following terms (“tuberculosis” OR “TB”) AND (“cause-specific mortality” OR “cause of death” OR “CoD”). Few studies evaluated long-term survival of newly-diagnosed patients. Most studies focused on short-term survival, and those that did look at long-term survival were mainly done in high-income countries. These studies described higher mortality in patients with tuberculosis compared with source populations or household controls. We found no detailed evaluations of cause-specific mortality nor any studies that evaluated the association between vulnerable conditions and cause-specific mortality with a competing risk framework for causes of death.

**Added value of this study**

In a middle-income country, we showed that the rate of death among patients with tuberculosis was six-times higher than the source population. This excess mortality was also observed among those who survived the first year of follow-up and was higher in patients with at least one vulnerable condition (eg, imprisonment, homelessness, alcohol and drug use, and diabetes). Infection was responsible for 45% of deaths. Homelessness, alcohol and drug use, and diabetes were associated with all-cause mortality. We have added to the literature showing that vulnerable conditions are associated with cause-specific mortality; homelessness and alcohol and drug use were associated with death from infection, respiratory, cardiovascular, and external or ill-defined causes, while diabetes was associated with deaths from cardiovascular causes.

**Implications of all the available evidence**

We showed the value of long-term follow-up of patients with tuberculosis, providing data to support a better understanding of tuberculosis burden, particularly after treatment. Vulnerable conditions are the main determinants of tuberculosis epidemics and treatment outcomes, and we observed that they are associated with cause-specific mortality. Two direct implications from our study are that mortality surveillance systems for long-term follow-up are needed and preventive measures for patients with vulnerable conditions should be reinforced. These measures could include reinforcing pneumococcal and influenza vaccination uptake, intensifying cardiovascular event prevention, a programme to reduce alcohol consumption, and tackling devastating conditions, such as homelessness.

## Methods

### Study design and participants

We did a retrospective, longitudinal study with data from the São Paulo State Tuberculosis Program, Brazil. São Paulo state is divided into 645 municipalities and the national public health system covers tuberculosis diagnosis and treatment free of charge. São Paulo state had a tuberculosis incidence of 39·1 per 100 000 inhabitants in 2010, and the highest absolute number of tuberculosis cases in Brazil.^14^, ^15^

We had official written permission for use of the data from the data guardian (the Health Department of São Paulo state) and ethics approval from the local ethics committee (protocol 270/14). The data for the general population is publicly available and did not require ethics approval.

We included patients with newly-diagnosed tuberculosis, aged 15 years or older, and notified to the São Paulo State Tuberculosis Program in 2010. We defined newly diagnosed as those who had never been treated for tuberculosis or who had taken anti-tuberculosis drugs for 1 month or less.^16^ We excluded patients whose diagnosis had changed during the follow-up period (ie, they did not have tuberculosis) and patients who had multidrug-resistant (MDR) tuberculosis.^16^ Patients without antibiotic susceptibility testing were assumed to not have MDR tuberculosis, considering the low prevalence of MDR disease in São Paulo state (<1%).^1^, ^17^ The source population (São Paulo state) comprised 41 million inhabitants (around 22% of the Brazilian population), most of whom lived in urban areas with marked indexes of inequality and poverty in 2010 (appendix p 3).^14^

### Procedures

All data sources used in this study are described in the appendix (p 4). We selected the population with tuberculosis from the dedicated electronic system TBweb.^14^, ^18^ This dedicated platform includes all notified cases of bacteriologically confirmed or clinically diagnosed tuberculosis, as defined by WHO,^16^ from residents in São Paulo state. Tuberculosis notification is compulsory in Brazil and only notified cases can start treatment. TBweb receives continuous input regarding patient treatment status from health-care units responsible for patient care until the end of treatment.^14^ We included patients from Jan 1, 2010, to Dec 31, 2010 (dates of notification). Detailed descriptions of definitions and recoding for variables from TBweb are given in the appendix (pp 5–6).

We evaluated the long-term survival and causes of death from the Mortality Information System (SIM) located at the Health Department of São Paulo state,^19^ which contains causes of death for all residents (including deaths occurring inside and outside São Paulo state). Death certificates from SIM are carefully revised for standardisation and appropriate definitions, following WHO recommendations and using the ICD-10.^20^ We did a probabilistic record linkage followed by manual revision between the tuberculosis cohort and mortality database (appendix p 7).

We used the WHO ICD-10 hierarchy and standard grouping of causes of death.^20^ Our primary analysis used causes of death as defined by WHO chapters; we selected six causes of death as competing events of interest—infection, respiratory, neoplasia, cardiovascular, external causes, and ill-defined. We did two sensitivity analyses, regrouping the causes of death into five new groups, to be more specific and informative about tuberculosis and vulnerable conditions (tuberculosis, infections not related to tuberculosis, respiratory causes without infection, ischaemic heart or cerebrovascular disease, and external causes or ill-defined) and regrouping causes of death into two groups to explore the associated burden of tuberculosis (tuberculosis as the underlying cause of death and any mention of tuberculosis codes in other lines). These divisions were defined a priori during the design of this study. Further information and the exact codes used are shown in the appendix (p 8).

Using a theoretical model about vulnerability and tuberculosis (appendix p 9), we selected six exposures present in the tuberculosis literature as different constructs of vulnerability,^1^, ^3^, ^4^, ^14^ labelling them social vulnerability (homeless or prison inmates), health behaviours (alcohol and drug use), and comorbidities (biological vulnerability), with diabetes and mental illness.^3^, ^21^ We also combined homelessness and alcohol and drug use as they are frequently associated,^14^ generating two derived variables as follows: a binary variable representing the presence of at least of one these conditions and another variable with four levels adding each vulnerability (appendix p 5).

### Outcomes

Our primary objective was to estimate excess mortality over 5 years and within the group who survived the first year, compared with the source population. Our secondary objective was to estimate the excess mortality in patients with tuberculosis with vulnerable conditions (social vulnerability, health behaviours, and comorbidities). Finally, we estimated the association between patients' vulnerable conditions and all-cause and cause-specific mortality.

### Statistical analysis

We chose to run our study for 1 year because of the feasibililty of doing a probabalistic record linkage, allowing for manual review of all possible pairs and increasing sensitivity and specificity to ascertain long-term survival. Therefore, the sample size was pragmatic. We were aware of the yearly sample size of newly diagnosed patients and their short-term survival and, for most of the exposures of interest, the cohort sample size and number of events were greater than that requested for typical power (80%) and α errors (5%). Continuous variables are listed as mean (SD) and median (IQR), as appropriate. Counts are described as proportions.

We described all-cause 5-year survival and compared this with the expected survival of the underlying population (ie, São Paulo state population) and estimated standardised mortality ratios (SMR).^22^, ^23^ We calculated expected survival and mortality from lifetables for the São Paulo state population, obtained from the Brazilian Institute of Geography and Statistics, matched by age (by 1 year age bands), sex (male *vs* female), and calendar year (2010, 2011, 2012, 2013, 2014, and 2015). We estimated SMR for the whole population and among those with or without any of the six exposures of interest, over 5 years and after 1 year of starting tuberculosis treatment.

We evaluated the association between the six exposures and all-cause survival among the tuberculosis cohort using Cox proportional hazards models. We analysed all-cause survival over 5 years and split this into early (all-cause survival over the first year) and late (all-cause survival after the first year) categories. In a sensitivity analysis for the late models, we ran a Cox model starting at the date of treatment outcome. We used previous knowledge to build a directed acyclic graph and select potential confounding factors a priori (appendix p 10), then estimated the crude and age-adjusted and sex-adjusted association for each exposure. We fit a fully adjusted model, entering the six exposures concurrently and adjusting for potential confounders (age, sex, level of education, self-reported skin colour, immunosuppression from causes other than HIV infection, anatomical tuberculosis classification, and microbiological status). We adjusted for HIV status and place of diagnosis by stratification in the fully adjusted model, allowing for different baseline hazard rates between strata. We tested the proportional hazard assumption, assessing interactions with survival time and examining Schoenfeld residual plots. Diabetes had non-proportional hazards and we accommodated for this by allowing time-dependent effects with an interaction term with survival time. In a post-hoc analysis, we modelled for diabetes in the fully adjusted model with flexible parametric survival modelling. For the Cox models, the time axis was follow-up and patients were right censored, with the time of end of follow-up fixed as Aug 31, 2015 (appendix p 7).

We analysed cause-specific mortality with competing risk analysis, as a death from a cardiovascular cause precluded the occurrence of other causes of death. We illustrated the occurrence of different causes of death over time and derived cumulative incidence function curves. The association between exposure and cause-specific mortality was estimated by cause-specific hazard ratios (HRs).^24^ We fit the same fully adjusted model as for all-cause mortality and considered each cause of death as competing events.

We had missing values for five covariates and self-reported skin colour and education had around 20% missing values. We investigated the missingness pattern and did multiple imputation, generating ten imputed datasets, by fully conditional specification, allowing for non-linearity of the Cox model. We ran a sensitivity analysis of all-cause survival in the complete case dataset. A description of the multiple imputation is shown in the appendix (p 11). A description of our sensitivity analyses is shown in the appendix (p 12).

All analyses were done with Stata version 13.1. We followed STROBE guideline recommendations.

### Role of the funding source

The funder of the study had no role in study design, data collection, data analysis, data interpretation, or writing of the report. All authors had full access to all data in the study and had final responsibility for the decision to submit for publication.

## Results

In 2010, there were 19 252 notifications of tuberculosis cases. We excluded 550 cases as patients were younger than 15 years, 556 cases that were not tuberculosis, 2597 retreatments, and 48 cases of MDR tuberculosis, resulting in a final cohort of 15 501 patients with tuberculosis. We recorded baseline characteristics of the 15 501 patients with tuberculosis and demographic characteristics of the São Paulo state population (table 1). The mean age of patients with tuberculosis was 40 years (SD 15).

**Table 1:** Baseline characteristics

	**Entire cohort (n=15 501)**	São Paulo state population 2010*
**Age, years**†		
15–25	2930 (19%)	21%
>25–35	4055 (26%)	23%
>35–45	3247 (21%)	19%
>45–55	2605 (17%)	16%
>55–65	1527 (10%)	11%
>65	1118 (7%)	10%
**Sex**		
Female	4683 (30%)	52%
Male	10 818 (70%)	48%
**Self-reported skin colour**‡		
White	7129 (55%)	64%
Brown or mixed	3989 (31%)	29%
Black	1488 (12%)	6%
Asian or Indigenous	237 (2%)	1%
**Years of schooling**§		
Illiterate	521 (4%)	4%
1–3	1465 (12%)	..
4–7	4620 (38%)	..
8–11	4518 (37%)	..
12–14	798 (6%)	23%
≥15	379 (3%)	41%
**Incarcerated**		
Yes	1609 (10%)	<1%
No	13 892 (90%)	>99%
**Homeless**		
Yes	391 (3%)	..
No	15 110 (97%)	..
**Alcohol use**		
Yes	2053 (13%)	..
No	13 448 (87%)	..
**Drug use**		
Yes	1019 (7%)	..
No	14 482 (93%)	..
**Diabetes**		
Yes	880 (6%)	13%
No	14 621 (94%)	..
**Mental disorder**		
Yes	336 (2%)	..
No	15 165 (98%)	..
**HIV status**		
Negative	11 155 (72%)	..
Positive	1874 (12%)	<1%
Unknown	2472 (16%)	..
**Immunosuppression from causes other than HIV infection**		
Yes	113 (1%)	..
No	15 388 (99%)	..
**Anatomical classification**		
Pulmonary tuberculosis	12 458 (80%)	..
Pulmonary tuberculosis and extrapulmonary tuberculosis	409 (3%)	..
Extrapulmonary tuberculosis	2280 (15%)	..
Miliary or disseminated	354 (2%)	..
**Microbiological status**¶		
Positive	10 956 (78%)	..
Negative	3010 (22%)	..
**Place of diagnosis**‖		
Primary or outpatient care	9349 (61%)	..
Emergency or urgent care facility	3300 (22%)	..
Hospitalised	2465 (16%)	..
Upon autopsy	159 (1%)	..
**Treatment outcome**		
Treatment success	12 227 (79%)	..
Treatment failure	254 (2%)	..
Death	1247 (8%)	..
Loss to follow-up	1537 (10%)	..
Not evaluated	236 (2%)	..

* Data sources (appendix p 4) were the 2010 census (age, sex, education, and self-reported skin colour), the primary care database (diabetes), InfoPen (incarceration), and Notifiable Diseases Information System (HIV status). We did not find a population-based estimate for some variables and we could not redistribute the data for education from the 2010 census on all category levels present in the tuberculosis data.

† Missing data n=19 (<1%).

‡ Missing data n=2658 (17%).

§ Missing data n=3200 (21%).

¶ Missing data n=1535 (10%).

‖ Missing data n=228 (2%).

Over a period of 5 years from tuberculosis diagnosis, 2660 (17%) of 15 501 patients died. Among 15 342 patients who started treatment, the total follow-up time was 68 787·15 person-years (median 4·96, IQR 4·68–5·28), with a mortality rate of 36·36 (95% CI 34·96–37·81) per 1000 person-years (appendix p 13).

Compared with the São Paulo state population—matched by age, sex, and calendar years—the SMR over the 5 years of follow-up was 6·47 (95% CI 6·22–6·73) and 8·06 (7·56–8·58) for patients with vulnerable conditions. The SMR was highest for those aged 35–45 years and during the first year of follow-up (table 2). Among those who survived the first year, the overall SMR was 3·93 (95% CI 3·71–4·17) and was 5·25 (4·80–5·73) for patients with vulnerable conditions (table 2).

**Table 2:** SMRs over 5 years of follow-up of patients newly diagnosed with tuberculosis compared with the São Paulo state population, matched by age, sex, and calendar year

		**Among all patients who started treatment**			**Among those alive after 1 year**		
		Whole population (n=15 342)	Population without any of the six exposures* (n=10 182)	Population with at least one of the six exposures* (n=5160)	Whole population (n=14 011)	Population without any of the six exposures* (n=9347)	Population with at least one of the six exposures* (n=4664)
Overall		6·47 (6·22–6·73)	5·72 (5·43–6·02)	8·06 (7·56–8·58)	3·93 (3·71–4·17)	3·31 (3·06–3·57)	5·25 (4·80–5·73)
Stratified by age, years*							
	15–25	7·63 (6·28–9·19)	5·28 (4·07–6·75)	7·56 (5·56–10·06)	5·29 (4·07–6·75)	4·77 (3·37–6·55)	6·29 (4·11–9·22)
	>25–35	11·73 (10·47–13·09)	8·90 (7·62–10·33)	10·6 (8·95–12·47)	7·31 (6·22–8·52)	6·33 (5·04–7·86)	8·67 (6·88–10·79)
	>35–45	13·54 (12·44–14·72)	10·76 (9·58–12·05)	13·69 (12·04–15·50)	8·30 (7·34–9·35)	6·95 (5·84–8·21)	10·39 (8·70–12·32)
	>45–55	8·15 (7·49–8·84)	6·79 (6·07–7·58)	8·46 (7·44–9·58)	4·71 (4·15–5·31)	4·01 (3·36–4·74)	5·83 (4·85–6·96)
	>55–65	5·38 (4·88–5·92)	4·68 (4·12–5·28)	5·39 (4·58–6·30)	3·16 (2·73–3·63)	2·91 (2·41–3·49)	3·63 (2·86–4·55)
	>65	3·27 (2·99–3·58)	2·71 (2·43–3·00)	3·09 (2·59–3·67)	1·92 (1·68–2·20)	1·79 (1·52–2·10)	2·33 (1·80–2·98)
Stratified by sex							
	Female	7·33 (6·76–7·94)	6·59 (5·99–7·24)	10·31 (8·81–11·99)	4·25 (3·76–4·78)	3·67 (3·16–4·23)	6·62 (5·27–8·20)
	Male	6·23 (5·95–6·52)	5·42 (5·10–5·76)	7·71 (7·19–8·26)	3·85 (3·60–4·11)	3·19 (2·91–3·48)	5·05 (4·58–5·55)
Stratified by follow-up, year							
	1st	16·54 (15·66–17·45)	15·33 (14·31–16·41)	19·07 (17·43–20·83)	..	..	..
	2nd	4·45 (3·99–4·94)	3·91 (3·40–4·49)	5·56 (4·68–6·57)	..	..	..
	3rd	3·82 (3·39–4·28)	3·35 (2·87–3·88)	4·80 (3·98–5·75)	..	..	..
	4th	3·32 (2·93–3·76)	2·56 (2·14–3·03)	4·95 (4·10–5·91)	..	..	..
	5th	3·47 (3·04–3·93)	2·76 (2·31–3·28)	5·00 (4·11–6·04)	..	..	..
Stratified by previous treatment outcome							
	Treatment success	..	..	..	3·47 (3·25–3·70)	2·91 (2·67–3·17)	4·71 (4·26–5·20)
	Treatment failure	..	..	..	5·23 (3·60–7·34)	5·81 (3·45–9·19)	4·66 (2·61–7·69)
	Loss to follow-up or not evaluated	..	..	..	8·75 (7·59–10·03)	7·89 (6·47–9·53)	9·91 (8·06–12·07)

Data are SMRs (95% CI). SMR=standardised mortality ratio.

* The six exposures are incarceration, homelessness, alcohol and drug use, diabetes, and mental disorders.

We found an increase in the proportion of deaths by baseline characteristics in older (>65 years; 48%), illiterate (28%), and immunosuppressed patients (HIV positive; 39%; appendix p 14).

After linking TBweb with the mortality database, we observed that 320 (18%) of 1773 patients who were lost to follow-up or had unevaluated treatment outcomes died (appendix p 14). A considerable proportion of these patients died soon after loss to follow-up (appendix p 15), at a median of 14 months (IQR 5–33). 1055 (9%) of 12 227 patients with previous treatment success died a median of 24 months (IQR 11–39) after successful treatment.

The most frequent cause of death was infection (1197 [45%] of 2660 deaths), followed by respiratory (334 [13%] of deaths), neoplasia (307 [12%] of deaths), cardiovascular (280 [11%] of deaths), and external causes (174 [7%] of deaths; appendix p 16). After 1 year, respiratory, neoplasia, cardiovascular, and external causes of death exceeded infection in patients without HIV coinfection. In patients younger than 30 years, infection (150 [55%] patients) and external causes (49 [18%] patients) were responsible for most deaths (appendix p 17). Patients with tuberculosis had a different distribution of the top ten causes of death compared with the São Paulo State population (appendix p 18) and this difference was more pronounced among those with vulnerable conditions (appendix p 19).

We estimated the crude and age-adjusted and sex-adjusted associations between exposures and all-cause survival (appendix p 20). After fully adjusting for confounders, homelessness, alcohol and drug use, or a combination of these two factors were consistently associated with reduced survival among patients with tuberculosis for the different periods analysed (table 3). We did not observe an association between imprisonment or mental disorder and survival. Diabetes had a time-dependent effect on survival, with an increasing adjusted HR over the period analysed. A flexible parametric survival model confirmed the non-linear risk of death (appendix pp 21–22). We observed similar point estimates for all associations in the complete case dataset (appendix p 23) and considering the starting point for the date of treatment outcome (appendix p 24).

**Table 3:** Association between social vulnerability, health behaviours, and comorbidities and 5-year survival of patients newly diagnosed with tuberculosis

		**5-year survival (n=15 342)**				**1-year survival (n=15 342)**				**Among those alive at 1 year (n=14 011)**			
		Patients	Deaths	Adjusted HR (95% CI)*	p value	Patients	Deaths	Adjusted HR (95% CI)*	p value	Patients	Deaths	Adjusted HR (95% CI)*	p value
**Social vulnerability**													
Incarcerated		..	..	..	0·189	..	..	..	0·388	..	..	..	0·261
	No	13 736	2376 (17%)	1 (ref)	..	13 736	1274 (9%)	1 (ref)	..	12 462	1102 (9%)	1 (ref)	..
	Yes	1 606	125 (8%)	0·88 (0·73–1·07)	..	1606	57 (4%)	0·88 (0·67–1·17)	..	1549	68 (4%)	0·86 (0·66–1·12)	..
Homelessness		..	..	..	<0·0001	..	..	..	0·0009	..	..	..	0·0081
	No	14 966	2382 (16%)	1 (ref)	..	14 966	1263 (8%)	1 (ref)	..	13 703	1119 (8%)	1 (ref)	..
	Yes	376	119 (32%)	1·51 (1·25–1·83)	..	376	68 (18%)	1·54 (1·19–1·98)	..	308	51 (17%)	1·48 (1·11–1·97)	..
**Health behaviours**													
Alcohol use		..	..	..	<0·0001	..	..	..	0·0006	..	..	..	<0·0001
	No	13 311	1988 (15%)	1 (ref)	..	13 311	1070 (8%)	1 (ref)	..	12 241	918 (8%)	1 (ref)	..
	Yes	2031	513 (25%)	1·36 (1·22–1·51)	..	2031	261 (13%)	1·30 (1·12–1·52)	..	1770	252 (14%)	1·42 (1·22–1·66)	..
Drug use		..	..	..	0·030	..	..	..	0·302	..	..	..	0·026
	No	14 324	2318 (16%)	1 (ref)	..	14 324	1236 (9%)	1 (ref)	..	13 088	1082 (8%)	1 (ref)	..
	Yes	1018	183 (18%)	1·20 (1·02–1·41)	..	1018	95 (9%)	1·13 (0·90–1·41)	..	923	88 (10%)	1·31 (1·03–1·65)	..
**Comorbidities**													
Diabetes mellitus†		..	..	..	0·067	..	..	..	0·084	..	..	..	0·011
	No	14 472	2279 (16%)	1 (ref)	..	14 472	1233 (9%)	1 (ref)	..	13 239	1046 (8%)	1 (ref)	..
	Yes	870	222 (25%)	0·83 (0·67–1·02)	..	870	98 (11%)	0·83 (0·67–1·03)	..	772	124 (16%)	1·28 (1·06–1·56)	..
Mental disorder		..	..	..	0·439	..	..	..	0·147	..	..	..	0·649
	No	15 011	2421 (16%)	1 (ref)	..	15 011	1283 (9%)	1 (ref)	..	13 728	1138 (8%)	1 (ref)	..
	Yes	331	80 (24%)	1·09 (0·87–1·38)	..	331	48 (15%)	1·25 (0·93–1·68)	..	283	32 (11%)	0·92 (0·64–1·32)	..
**Combined**													
Alcohol or drug use or homelessness		..	..	..	<0·0001	..	<0·0001	..	<0·0001	..	..	..	<0·0001
	No	12 558	1852 (15%)	1 (ref)	..	12 558	998 (8%)	1 (ref)	..	11 560	854 (7%)	1 (ref)	..
	Yes	2784	649 (23%)	1·45 (1·32–1·60)	..	2784	333 (12%)	1·37 (1·20–1·57)	..	2451	316 (13%)	1·55 (1·35–1·78)	..
Alcohol and drug use and homelessness		..	..	..	..	..	..	..	..	..	..	..	..
	One factor	2199	500 (23%)	1·38 (1·25–1·54)	..	2199	253 (12%)	1·31 (1·13–1·51)	..	1946	247 (13%)	1·47 (1·27–1·71)	..
	Two factors	529	132 (25%)	1·73 (1·44–2·08)	..	529	69 (13%)	1·57 (1·22–2·02)	..	460	63 (14%)	1·97 (1·51–2·57)	..
	Three factors	56	17 (30%)	2·11 (1·30–3·42)	..	56	11 (20%)	2·36 (1·29–4·30)	..	45	6 (13%)	1·75 (0·77–3·93)	..

HR=hazard ratio.

* Fully adjusted models in ten multiple imputed datasets.

† Time-varying effect of diabetes mellitus interacting with survival time HR for the interaction term 1·15 (95% CI 1·05–1·25); p=0·0015.

Following tuberculosis diagnosis, the hazard rate of deaths due to infection reached a plateau and this effect was more apparent in those without HIV coinfection (appendix p 25).

Among patients with tuberculosis, homelessness, alcohol and drug use, or a combination of these factors were associated with deaths due to infection (cause-specific HR 1·60, 95% CI 1·39–1·85 for the combination), external (1·66, 1·18–2·33 for the combination), and ill-defined causes (2·06, 1·31–3·25 for the combination; figure; appendix p 26). Mental disorders were associated with respiratory (1·76, 1·09–2·85) and ill-defined causes (2·39, 1·00–5·67) of death. Diabetes (1·70, 1·23–2·35) and the combination of homelessness and alcohol and drug use (1·43, 1·06–1·95) were associated with cardiovascular deaths. A similar association was observed among those without immunosuppression (appendix pp 27–28).

**Figure:**
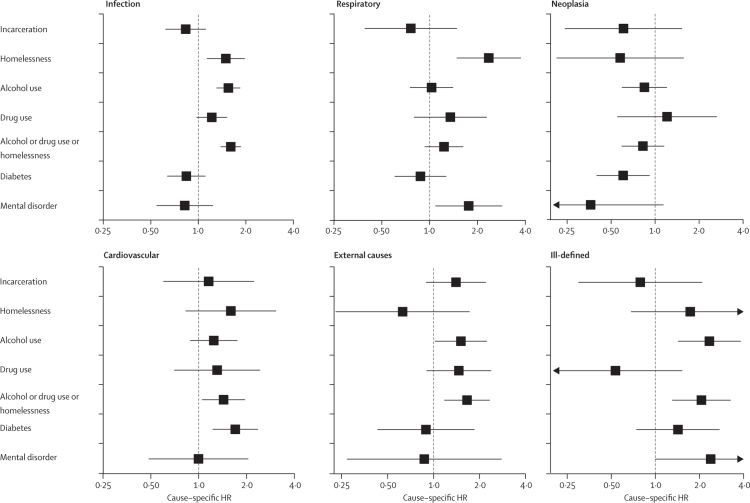
Fully adjusted cause-specific HRs for 5-year cause-specific mortality in patients newly diagnosed with tuberculosis Boxes show adjusted cause-specific HRs and error bars show 95% CIs. Arrowheads represents the direction of the CI value, truncated at the horizontal axis limit. HR=hazard ratio.

For cause-specific fully adjusted HRs for exposures among those who survived the first year we observed an association between the combination of homelessness and alcohol and drug use and all causes of death, except neoplasia, and diabetes with cardiovascular causes (appendix pp 29–30). After 1 year of follow-up, prison incarceration was associated with external causes of death (cause-specific HR 1·64, 95% CI 0·98–2·74).

Cumulative incidence curves for the first regrouping of causes of death are shown in the appendix (p 31). The homelessness and alcohol and drug use combination was associated with all causes of death (HR 1·80, 95% CI 1·37–2·36 for external or ill-defined causes), except respiratory causes (not pneumonia), over 5 years (appendix pp 32–35) and after the first year (appendix pp 36–37). The combination of homelessness and alcohol and drug use had the highest point estimate for external or ill-defined causes (cause-specific HR 1·89, 95% CI 1·36–2·62), followed by tuberculosis (1·82, 1·06–3·12) after 1 year.

Cumulative incidence curves for deaths attributed to tuberculosis are shown in the appendix (p 38). 531 (20%) of 2660 total patients who died had tuberculosis as the underlying cause of death and 366 (14%) had tuberculosis mentioned in other lines of their death certificate. When we analysed deaths during the first year, 462 (31%) of 1490 patients who died had tuberculosis as the underlying cause of death and 294 (20%) had tuberculosis mentioned in other lines of their death certificate; these proportions were 69 (6%) of 1170 and 72 (6%) of 1170, respectively, among those who survived after the first year. The homelessness and alcohol and drug use combination was associated with death with any mention of tuberculosis over 5 years (appendix pp 39–40) and after 1 year (cause-specific HR 1·83, 95% CI 1·26–2·66; appendix pp 41–42).

## Discussion

In this population-based study in São Paulo state, Brazil, we observed that 2660 (17%) of 15 501 patients with tuberculosis died within 5 years after tuberculosis diagnosis. Mortality among patients with tuberculosis was six-times higher than the source population, and four-times higher for those who survived the first year. Infection was responsible for most deaths. Homelessness, alcohol and drug use, and diabetes were associated with lower survival among patients with tuberculosis. These vulnerable conditions were associated with cause-specific mortality such as death from tuberculosis, from external or ill-defined causes, and cardiovascular causes.

In 2017, as estimated by WHO, there were around 1·6 million tuberculosis deaths.^1^ Tuberculosis is a treatable disease; therefore, deaths and tuberculosis-associated sequelae should be avoidable.^1^, ^13^ Accurate estimation of the tuberculosis attributable burden is fundamental to guide public health programmes and prioritise global policy decisions.^1^, ^25^, ^26^ A strength of this study is evaluation of the long-term survival of patients with tuberculosis in a high-burden country, with application of competing risk analysis to study cause-specific mortality among patients with tuberculosis, and using data from death certificates from an established vital registration system.

We observed that patients with tuberculosis died at a higher rate than the source population over 5 years of follow-up, similar to findings previously reported in India (SMR 6·1; for 2000–03).^10^ When considering those who survived the first year, we observed a SMR comparable to that reported in post-treatment cohorts in Israel (3·7; for 2000–10),^7^ and Vietnam (4·0; for 2010–13).^11^ Although this study is restricted by not using individual data to build a cohort without tuberculosis and not disentangling excess mortality from tuberculosis itself or vulnerable conditions, we observed that patients with tuberculosis with vulnerable conditions had even higher excess mortality (SMR 8·1 over 5 years, and 5·3 for those who survived the first year), compared with the source population. The highest SMRs were for those aged 20–40 years,^10^, ^12^ showing how tuberculosis affects a working age population, and indicating the need to better evaluate the catastrophic costs associated with tuberculosis after treatment.^27^

The main reason for several countries, including Brazil, not achieving the WHO treatment outcome goal is loss to follow-up.^1^, ^14^ After linkage with the mortality database, we observed that patients who had initially been declared lost to follow-up in the treatment database actually died soon after. This result reinforces the recommendation that national programmes should focus on high-risk groups to decrease their risk of loss to follow-up. Additionally, our findings reiterate the importance of record linkage and monitoring of post-tuberculosis treatment mortality, to provide more accurate data for national programmes and burden estimation.^1^, ^25^, ^26^

The combination of homelessness and alcohol and drug use among patients with tuberculosis was associated with every cause-specific mortality we investigated except neoplasia. Previous studies showed that these conditions were associated with all-cause mortality,^3^ particularly alcohol consumption, in high-income countries^12^, ^28^ and low-income and middle-income countries.^10^ Although not estimated by a quantitative measure of association (eg, cause-specific HR), studies done in Israel^7^ and Denmark^12^ with death certificate data, together with the study from Vietnam,^11^ which used verbal autopsy, reported a higher proportion of deaths probably associated with alcohol and drug use among patients with tuberculosis compared with the source population. We observed that, among patients with tuberculosis, lower acute respiratory infections and chronic respiratory diseases were the top causes of death, which is compatible with evidence showing post-tuberculosis lung sequelae.^13^ 120 successfully treated patients with tuberculosis were evaluated for lung function in Brazil in 2013–14 and alcohol use was one of the factors associated with worse lung function,^29^ highlighting that this population might benefit from already well known and simple interventions, such as pneumococcal and influenza vaccines, and a programme to reduce alcohol consumption.

Diabetes is a key comorbidity in the tuberculosis epidemic,^1^ and tuberculosis was ranked as the first cause of death among patients with tuberculosis who had diabetes. We observed that diabetes was non-linearly associated with all-cause mortality, starting from a protective association to a smoothed increase in risk over time. This pattern disappeared for cause-specific mortality, when diabetes was consistently associated with cardiovascular deaths. Our results are in accordance with a study in Mexico (from 1995–2010), which observed worse intermediate outcomes during treatment, but no increased risk of tuberculosis-related mortality for patients with diabetes.^30^ We had no information about diabetes severity or glycaemic control for patients with tuberculosis, factors that could help explain the complex association between diabetes, treatment outcomes, and all-cause mortality.

Imprisonment is a particularly vulnerable condition. We observed that imprisonment was not associated with worse all-cause survival, but imprecisely associated with external causes of death after the first year of follow-up. The presence of other vulnerable factors (eg, tuberculosis and HIV coinfection and alcohol use) could explain part of this finding,^31^ associated with substantial collaborative efforts of the São Paulo State Tuberculosis Program to improve treatment outcomes for tuberculosis cases that occur in prisons.^15^

This study has limitations that must be acknowledged. First, we used a population-based cohort, and because notification is compulsory and needed for medication delivery in Brazil, it is unlikely we have missed a relevant number of cases that started treatment (not considering cases diagnosed after death). However, routine databases have intrinsic limitations—not all patients were tested for HIV or anti-tuberculosis drug resistance. However, HIV testing was relatively high^1^ and we allowed a baseline hazard for each HIV status in the Cox models. Regarding MDR tuberculosis, Brazil and São Paulo have historically low proportions of MDR disease among new cases (<1% in 2010);^17^ this proportion was maintained after the ruling out of rapid diagnostic tests,^1^ so we did not expect much bias from possible misclassification. Second, the sample size was pragmatically chosen to be feasible for probabilistic linkage with manual review. This factor might have affected our power to detect some associations for cause-specific mortality. Additionally, the performance of the probabilistic linkage was excellent, but estimated with the mortality data entered into TBweb. Thus, the linkage procedure could have lower sensitivity than estimated. We guaranteed high specificity by manually reviewing all pairs selected in the probabalistic record linkage. Third, to ascertain a single cause of death is arbitrary and we overcame this by sensitivity analyses. Fourth, point estimates could be overestimated because of positive residual confounding—we had no data about smoking or nutritional status. Additionally, some of our exposures had no quantitative or cumulative information, such as alcohol consumption. Finally, we excluded patients who had been retreated for tuberculosis, who are associated with vulnerable conditions and lower survival,^1^, ^3^ thereby limiting our generalisability to all patients with tuberculosis and underestimating the total disease burden.

Our study provides an extensive epidemiological analysis of long-term survival and cause-specific mortality of tuberculosis in a high-burden country, showing that this disease makes a notable contribution to excess mortality and its association with vulnerable conditions. Policy makers, society stakeholders, community members, and health professionals should concentrate efforts to tackle tuberculosis and its association with various vulnerable conditions.
