# Supplementary material for: Long-term survival and cause-specific mortality of patients newly diagnosed with tuberculosis in São Paulo state, Brazil, 2010–15: a population-based, longitudinal study
Source: Lancet Infect Dis. 2020 Jan;20(1):123–32. doi: 10.1016/S1473-3099(19)30518-3 (PMC6928568; doi:10.1016/S1473-3099(19)30518-3)
Supplement: Supplementary appendix [file mmc1.pdf]

THE LANCET Infectious Diseases

Supplementary webappendix

This webappendix formed part of the original submission and has been peer reviewed. We post it as supplied by the authors.

Supplement to: Ranzani OT, Rodrigues LC, Bombarda S, MintoCM, Waldman EA, Carvalho CRR. Long-term survival and cause-specific mortality of patients newly diagnosed with tuberculosis in São Paulo state, Brazil, 2010–15: a population-based, longitudinal study. *Lancet Infect Dis* 2019; published onlined Oct 29. https://doi.org/10.1016/S1473-3099(19)30518-3.

Supplementary appendix

Table of Contents

eTable 1. General characteristics of the tuberculosis cohort and the São Paulo State population in 2010...3

eTable 2. Description of the data sources used in this study..4

eTable 3. Detailed description of variables used in the study 5

eTables 4. Adapted from the 2013 WHO Definitions and reporting framework for tuberculosis – 2013 revision (updated December 2014)[5]...6

Supplementary methods: Linkage 7

eTable 5: List of the International Classification of Diseases 10th revision (ICD) codes used in this study for causes of death...8

eFigure 1. Theoretical model used to support the research questions regarding vulnerability and TB 9

eFigure 2. Direct acyclic graph (DAG) for the association between different vulnerabilities (inmate, homelessness, alcohol and drug use, diabetes mellitus and mental disorder) and survival of newly-diagnosed tuberculosis patients. 10

Supplementary methods: Missing data / Multiple imputation..**11**

eTable 6.A. Frequency of missing values in the five covariates who were imputed 11

eTable 6.B. Methods used to impute the five covariates 11

eTable 6.C. Frequency distribution of the five imputed covariates before and after imputation................11

eTable 7. Description of sensitivity analyses...12

eFigure3. Long-term survival of tuberculosis patients newly-diagnosed in 2010 in São Paulo State, Brazil. 13

eTable 8. The crude mortality observed over 5-years of follow-up of newly-diagnosed tuberculosis cases stratified by characteristics at baseline and treatment outcome 14

eFigure4. Cumulative hazard of mortality stratified by treatment outcome 15

eFigure 5. Causes of death of newly-diagnosed tuberculosis patients stratified by TB-HIV coinfection status and time of follow-up...16

eFigure 6. Causes of death of newly-diagnosed tuberculosis cases, stratified by age and time from diagnosis..17

eTable 9. Top 10 causes of death during 5-year follow-up of tuberculosis patients newly diagnosed in 2010 in São Paulo State, Brazil. 18

eTable 10. Top 10 causes of death during 5-year follow-up of newly diagnosed tuberculosis patients stratified by the exposures of interest. 19

eTable 11. Crude and age-sex adjusted hazard ratios for all-cause 5-year survival, 1-year survival and among those who survived the first year. 20

eTable 12. The association between social vulnerability, health behaviours, and comorbidities and 5-year survival of newly-diagnosed tuberculosis patients in fully adjusted models, fitting a Cox proportional hazard model with time-dependent effect for diabetes mellitus and a flexible parametric survival model. 21

eFigure 7. Fully adjusted hazard ratio for diabetes mellitus from the parametric flexible survival model over 5-years from diagnosis. 22

eTable 13. Sensitivity analysis for fully adjusted models for all-cause survival while using the multiple imputation or complete case analysis...23

eTable 14. Sensitivity analysis for the association between vulnerable conditions and all-cause mortality considering starting the follow-up time after 1 year or after treatment definition occurrence 24

eFigure 8. Cumulative incidence function for 5 and 1-year cause-specific mortality stratified by TB-HIV coinfection status. 25

eTable 15. The association between social vulnerability, health behaviours, and comorbidities and 5-year cause-specific mortality of tuberculosis patients newly-diagnosed in 2010 in São Paulo State, Brazil (Population: Whole cohort)....................26

eTable 16. The association between social vulnerability, health behaviours, and comorbidities and 5-year cause-specific mortality of tuberculosis patients newly diagnosed in 2010 in São Paulo State, Brazil (Population: Not immunocompromised cohort). 27

eFigure 9. Fully adjusted cause-specific hazard ratios for 5-year cause-specific mortality (Population: Not immunocompromised cohort)....................28

eTable 17. The association between social vulnerability, health behaviours, and comorbidities and 5-year cause-specific mortality among those patients who survived the 1 year in 2010 in São Paulo State, Brazil. 29

eFigure 10. Fully adjusted cause-specific hazard ratios for 5-year cause-specific mortality (Population: Among those who survived the first year) 30

eFigure 11. Cumulative incidence function for 5 and 1-year cause-specific mortality (sensitivity analysis for cause of death) 31

eTable 18. The association between social vulnerability, health behaviours, and comorbidities and 5-year cause-specific mortality of tuberculosis patients newly diagnosed in 2010 in São Paulo State, Brazil (sensitivity analysis for cause of death). 32

eTable 19. The association between social vulnerability, health behaviours, and comorbidities and 5-year cause-specific mortality of tuberculosis patients newly diagnosed in 2010 in São Paulo State, Brazil (sensitivity analysis for causes of death. Population: Not immunocompromised cohort)....................33

eFigure 12. Fully adjusted cause-specific hazard ratios for 5-year cause-specific mortality (sensitivity analysis for causes of death)....................34

eFigure 13. Fully adjusted cause-specific hazard ratios for 5-year cause-specific mortality (sensitivity analysis for causes of death. Population: Not immunocompromised cohort) 35

eTable 20. Fully adjusted cause-specific hazard ratios for 5-year cause-specific mortality among those patients who survived the first year in 2010 in São Paulo State, Brazil (sensitivity analysis for cause of death)....................36

eFigure 14. Fully adjusted cause-specific hazard ratios for 5-year cause-specific mortality among those patients who survived the first year in 2010 in São Paulo State, Brazil (sensitivity analysis for cause of death)....................37

eFigure 15. Cumulative incidence function for 5 and 1-year TB related cause-specific mortality stratified by TB-HIV coinfection status. 38

eTable 21. The association between social vulnerability, health behaviours, and comorbidities and 5-year mortality related to any mention to tuberculosis in the death certificate of tuberculosis patients newly diagnosed in 2010 in São Paulo State, Brazil (sensitivity analysis for causes of death 2. Population: Whole and not immunocompromised cohorts). 39

eFigure 16. Fully adjusted cause-specific hazard ratios for 5-year tuberculosis related cause-specific mortality (sensitivity analysis for causes of death-2. Population: Whole and not immunocompromised cohorts)....................40

eTable 22. The association between social vulnerability, health behaviours, and comorbidities with 5-year mortality related to any mention to tuberculosis in the death certificate among those patients who survived the 1 in 2010 in São Paulo State, Brazil (sensitivity analysis for cause of death - 2)....................41

eFigure 17. Fully adjusted cause-specific hazard ratios for 5-year tuberculosis related cause-specific mortality among those alive after the 1-year (sensitivity analysis for causes of death-2) 42

REFERENCES....................43

eTable 1. General characteristics of the tuberculosis cohort and the São Paulo State population in 2010.

Variable	Values	Whole cohort (n = 15501)	São Paulo State 2010*
**Age**, years[a]	15-25	2930 (18·9%)	21·4%
	25·1-35	4055 (26·2%)	22·7%
	35·1-45	3247 (21·0%)	19·0%
	45·1-55	2605 (16·8%)	16·0%
	55·1-65	1527 (9·9%)	10·9%
	>65·1	1118 (7·2%)	10·0%
**Sex**	Female	4683 (30·2%)	51·9%
	Male	10818 (69·8%)	48·1%
**Self-reported skin colour** [b]	White	7129 (55·5%)	63·9%
	Brown/Mixed	3989 (31·1%)	29·1%
	Black	1488 (11·6%)	5·5%
	Asian/Indigenous	237 (1·9%)	1·5%
**Level of education,** number of years of schooling [c]	Illiterate	521 (4·2%)	4·3%
	1-3 years	1465 (11·9%)	
	4-7 years	4620 (37·6%)	
	8-11 years	4518 (36·7%)	
	12-14 years	798 (6·5%)	23·0%
	≥15 years	379 (3·1%)	40·6%
**Inmate**	Yes	1609 (10·4%)	0·5%
**Homelessness**	Yes	391 (2·5%)	
**Alcohol use**	Yes	2053 (13·2%)	
**Drug users**	Yes	1019 (6·6%))	
**Diabetes mellitus**	Yes	880 (5·7%)	12·6%
**Mental disorder**	Yes	336 (2·2%)	
**HIV status**	Negative	11155 (72·0%)	
	Positive	1874 (12·1%)	<1·0%
	Unknown	2472 (15·9%)	
**Immunosuppression from aetiologies other than HIV infection**	Yes	113 (0·7%)	
**Population**			41,262,199
**Population ≥ 15 years**			32,401,281
**Municipalities**			645
**Proportion of population on urban areas**			95·9%
**Life expectancy**			75·7 years
**Gini index**			0·56
**Human development index**			0·783
**Municipal – Human development index** (min-max)			0·639-0·862

[a] Missing data: n = 19 (0·1%); [b] Missing data: n = 2658 (17·2%); [c] Missing data: n = 3200 (20·6%); [d] Missing data: n = 1535 (9·9%); [e] Missing data: n = 228 (1·5%). PTB: pulmonary tuberculosis; EPTB: extrapulmonary tuberculosis.

* Data sources (references below): Census 2010 (age, sex, education, self-reported skin-colour, population, urbanization); Primary Care Database (SiAB): diabetes mellitus; InfoPen: inmates; SINAN: HIV status; Brazilian Atlas (life expectancy, Gini index, human development index); SEADE (municipal human development index). For the demographic missing variables for the São Paulo State population, we did not find a population-based estimate and for education, we could not re-distribute the data from the Census 2010 on all category levels present on the tuberculosis data.

eTable 2. Description of the data sources used in this study.

Data	Source	Data	Date exported / Checked
Tuberculosis cohort	TBweb[1], São Paulo State Health Department	The data cannot be publicly shared since it contains sensitive patient information and due to ethical restrictions imposed by the Health Department in São Paulo State, Brazil. The data could be requested from: http://www.saude.sp.gov.br/cve-centro-de-vigilancia-epidemiologica-prof.-alexandre-vranjac/areas-de-vigilancia/tuberculose/ or by email: dvtbc@saude.sp.gov.br.	14/09/2014
Mortality data	SIM, São Paulo State Health Department	The data cannot be publicly shared since it contains sensitive patient information and due to ethical restrictions imposed by the Health Department in São Paulo State, Brazil. The data could be requested from: http://www.saude.sp.gov.br/coordenadoria-de-controle-de-doencas/homepage/destaques/simsinasc or by email: civs.ccd@saude.sp.gov.br	18/12/2015
São Paulo State Life Tables	IBGE, Brazil, 2010-2016	https://www.ibge.gov.br/estatisticas/sociais/populacao/9109-projecao-da-populacao.html?=&t=downloads<br>ftp://ftp.ibge.gov.br/Projecao_da_Populacao/Projecao_da_Populacao_2018/Tabuas_Mortalidade%202010-2060.xls	19/05/2019
São Paulo State general characteristics in 2010 (age, education and self-reported skin colour)	Censo 2010, IBGE	ftp://ftp.ibge.gov.br/Censos/Censo_Demografico_2010/Educacao_e_Deslocamento/xls/sao_paulo_xls.zip<br>ftp://ftp.ibge.gov.br/Censos/Censo_Demografico_2010/resultados_preliminares/Tabela4.zip<br>ftp://ftp.ibge.gov.br/Censos/Censo_Demografico_2010/resultados_preliminares/Tabela9.zip<br>http://www.atlasbrasil.org.br/2013/pt/perfil_uf/sao-paulo	19/05/2019
São Paulo State general characteristics (diabetes mellitus registred in primary care in 2010)	Brazilian National Ministry of Health Department of Primary Care database, SiAB - SP, 2010	http://tabnet.datasus.gov.br/cgi/deftohtm.exe?siab/cnv/SIABSSP.def	15/05/2019
Inmates population in 2010	Penitentiary Department, Ministry of Justice, InfoPen	http://depen.gov.br/DEPEN/depen/sisdepen/infopen/relatorios-sinteticos/populacaocarcerariasintetico2010.pdf	15/05/2019
HIV positive notifications in 2010	National Notification System, SINAN	http://www.aids.gov.br/system/tdf/pub/2016/59427/boletim_2016_1_pdf_16375.pdf	15/05/2019

eTable 3. Detailed description of variables used in the study

	Variable	Original coding	Collected from	Recoding for this study	Comments
**Exposures**	Imprisonment	2 levels	Official status	None	For those who were incarcerated, including those in penal institutions awaiting trial or sentencing, at notification or when starting TB treatment.
	Homelessness	2 levels	Self-reported/address	None	Homelessness was considered for those without a fixed, regular, and adequate night-time residence. This definition includes individuals who live in emergency shelters/direct access hostels and those who live in places not meant for human habitation. Defined at notification or when starting TB treatment.
	Alcohol	2 levels	Clinical record/Self-reported	None	Related to current any alcohol use at notification at notification or when starting TB treatment.
	Drug users	2 levels	Clinical record/Self-reported	None	Related to current use of any illicit drug at notification or when starting TB treatment.
	Diabetes mellitus	2 levels	Clinical record/Self-reported	None	Diabetes mellitus type 1 or 2 at notification or when starting TB treatment,
	Mental disorder	2 levels	Clinical record/Self-reported	None	Any mental disorder as defined by the International Code of Disease-ICD at notification or when starting TB treatment.
	Combined vulnerabilities	2 levels (binary) or 4 levels (0, 1, 2, 3)	Derived	Combination of Homelessness or Alcohol or Drug use as a 2 levels variable. Also added Homelessness and Alcohol and Drug use generating a 4 levels variable: 0, 1, 2 or 3 factors	Recoded because of the frequent overlap between these exposures[2]
**Covariates**	Age	12 levels (8 levels for those above 15 y)	National ID	Recode from 8 to 6 levels, collapsing the categories of those above 65 y	Recoded to avoid sparse data for those above 65 y
	Sex	2 levels	National ID	None	
	Self-reported skin colour	5 levels	Self-reported	Combined Asian and Indigenous	Self-reported skin colour is an important surrogate for socioeconomic position, social inequality, social capital, and social inclusion in Brazil[3].
	Education	6 levels	Self-reported	None	Self-reported and categorized in 6 levels of years of education
	HIV status	3 levels	Laboratory evaluation	None	WHO definition (below). The HIV status could be updated during treatment.
	Other immunosuppressio	2 levels	Clinical record/Self-reported	None	Transplanted patients, in use of immunosuppressant drugs and chemotherapy at notification or when starting TB treatment.
	Place of diagnosis	7 levels	Place of Notification	Recoded to 3 categories: Primary care (included ambulatory, active case finding in community or prisons, and contact tracing); Emergency (urgency/emergency facilties) and Hospitalized (hospitals). The category “after death” was excluded in the survival analysis.	Primary care was recoded because of not being in hospital settings, such as emergence/urgency facilities or during hospitalization.
	Anatomical classification	14 levels (affected organs)	Clinical, laboratory and chest-X-ray evaluation	Recoded to 4 levels: Pulmonary only, Pulmonary and any concomitant extrapulmonary sites and combinations, Extrapulmonary only and Miliary/Disseminated (disseminated defined as occurrence of two or more sites not including lung parenchyma and/or a positive blood culture).	Description and justification described previously[4].
	Microbiologic status at diagnosis	4 levels	Sputum/Culture at diagnosis	Combination of a positive results on sputum-smear, other sample smear, or respiratory or other tissue culture. Recoded as positive or negative, and collapsed “requested”/”not done” as missing.	WHO definition (below)

eTables 4. Adapted from the 2013 WHO Definitions and reporting framework for tuberculosis – 2013 revision (updated December 2014)[5]

**Definition**	**Explanation**
**Case definitions - A.1**	
Bacteriologically confirmed TB case	is one from whom a biological specimen is positive by smear microscopy, culture or WHO-approved rapid diagnostics (such as Xpert MTB/RIF). All such cases should be notified, regardless of whether TB treatment has started
Clinically diagnosed TB case	is one who does not fulfil the criteria for bacteriological confirmation but has been diagnosed with active TB by a clinician or other medical practitioner who has decided to give the patient a full course of TB treatment. This definition includes cases diagnosed on the basis of X-ray abnormalities or suggestive histology and extrapulmonary cases without laboratory confirmation. Clinically diagnosed cases subsequently found to be bacteriologically positive (before or after starting treatment) should be reclassified as bacteriologically confirmed
**Classification based on history of previous TB treatment – item A.1.2**	
New patients	have never been treated for TB or have taken anti-TB drugs for less than 1 month
**Classification based on HIV status – item A.1.3**	
HIV-positive TB patient	refers to any bacteriologically confirmed or clinically diagnosed case of TB who has a positive result from HIV testing conducted at the time of TB diagnosis or other documented evidence of enrolment in HIV care, such as enrolment in the pre-ART register or in the ART register once ART has been started
HIV-negative TB patient	refers to any bacteriologically confirmed or clinically diagnosed case of TB who has a negative result from HIV testing conducted at the time of TB diagnosis. Any HIV-negative TB patient subsequently found to be HIV-positive should be reclassified accordingly
HIV status unknown TB patient	refers to any bacteriologically confirmed or clinically diagnosed case of TB who has no result of HIV testing and no other documented evidence of enrolment in HIV care. If the patient's HIV status is subsequently determined, he or she should be reclassified accordingly
**Classification based on drug resistance – item A.1.4**	
Multidrug resistance	resistance to at least both isoniazid and rifampicin.
**TB Outcomes from WHO (not includes resistant TB) – item A.2.1**	
Treatment success	The sum of cured and treatment completed
Cured	A pulmonary TB patient with bacteriologically confirmed TB at the beginning of treatment who was smear- or culture-negative in the last month of treatment and on at least one previous occasion
Completed treatment	A TB patient who completed treatment without evidence of failure but with no record to show that sputum smear or culture results in the last month of treatment and on at least one previous occasion were negative, either because tests were not done or because results are unavailable
Treatment failed*	A TB patient whose sputum smear or culture is positive at month 5 or later during treatment.
Died	A TB patient who dies for any reason before starting or during the course of treatment
Lost to follow-up	A TB patient who did not start treatment or whose treatment was interrupted for 2 consecutive months or more
Not evaluated	A TB patient for whom no treatment outcome is assigned. This includes cases "transferred out" to another treatment unit as well as cases for whom the treatment outcome is unknown to the reporting unit

*For the Brazilian and São Paulo State TB Program, treatment failure was originally defined as any proven resistance to TB drugs during the treatment. For this study we derived the treatment failure as described by the WHO using monthly sputum smear or culture results.

Supplementary methods: Linkage

Linkage between TB cohort and long-term survival/cause of death: To ascertain survival status and cause of death, we conducted a probabilistic record linkage between the TB cases notified in 2010 (n=15,501) and the mortality database (restricted to those aged ≥15 years and from 01/01/2010 to 31/08/2015, n=1,048,256). The probabilistic record linkage was conducted following the standard recommended steps.[6] Briefly, we performed the pre-merge data cleaning and standardisation, using the software RecLink-III (Universidade Federal do Rio de Janeiro, Rio de Janeiro, Brazil), which is adapted for Brazilian names.[7] We applied a validated system for the probabilistic linkage, composed by: A) Matching variables: patient's name and date of birth, and patient's mother name; B) Blocking variables: patient's first and last names (as Soundex codes) and sex; C) Auxiliary variable while manually reviewing: patient's address.[8,9] The probabilistic record linkage was conducted using the software Link Plus (Centers for Disease Control and Prevention, USA). Following the probabilistic linkage, we manually revised all record pairs with a non-zero weight to identify true-pairs. We conducted all the steps blinded from the known survival status registered in the TBweb database, covering the treatment period. For sensitivity estimation, we cross-tabulated deaths on TBweb ("gold standard") with deaths from the SIM (a case labelled "died" on TBweb had to be found on SIM, independent of any time-frame). For specificity, we considered cases linked by SIM when the date of death was within the time frame of treatment follow-up registered on the TBweb, ie, before the last follow-up on TBweb. For instance, if a hypothetical patient started treatment on 20/01/2010 and was declared as "treatment success" on TBweb on 26/08/2010, but we could link this patient and discover he/she died on 22/12/2010, the patient was declared alive for the linkage performance evaluation. The linkage procedure had a sensitivity of 96% and specificity of 99% compared to the known deaths registered in TBWeb during the treatment period.

eTable 5: List of the International Classification of Diseases 10th revision (ICD) codes used in this study for causes of death.

Primary analysis	Codes	Exactly as WHO classification in Chapters and Blocks	Used only underlying cause of death
**Infection** (Chapter I: Certain infectious and parasitic diseases)	A00-A09, A15-A19, A20-A28, A30-A49, A50-A64, A65-A69, A70-A74, A75-A79, A80-A89, A92-A99, B00-B09, B15-B19, B20-B24, B25-B34, B35-B49, B50-B64, B65-B83, B85-B89, B90-B94, B95-B98, B99.	Yes	Yes
**Respiratory** (Chapter X: Diseases of the respiratory system)	J00-J06, J09-J18, J20-J22, J30-J39, J40-J47, J60-J70, J80-J84, J85-J86, J90-J94, J95-J99.	Yes	Yes
**Neoplasia** (Chapter II: Neoplasms)	C00-C14, C15-C26, C30-C39, C40-C41, C43-C44, C45-C49, C50-C50, C51-C58, C60-C63, C64-C68, C69-C72, C73-C75, C76-C80, C81-C96, C97-C97, D00-D09, D10-D36, D37-D48.	Yes	Yes
**Cardiovascular** (Chapter IX: Diseases of the circulatory system)	I00-I02, I05-I09, I10-I15, I20-I25, I26-I28, I30-I52, I60-I69, I70-I79, I80-I89, I95-I99.	Yes	Yes
**External causes** (Chapter XX: External causes of morbidity and mortality)	V01-V09, V10-V19, V20-V29, V30-V39, V40-V49, V50-V59, V60-V69, V70-V79, V80-V89, V90-V94, V95-V97, V98-V99, W00-W19, W20-W49, W50-W64, W65-W74, W75-W84, W85-W99, X00-X09, X10-X19, X20-X29, X30-X39, X40-X49, X50-X57, X58-X59, X60-X84, X85-Y09, Y10-Y34, Y35-Y36, Y40-Y59, Y60-Y69, Y70-Y82, Y83-Y84, Y85-Y89, Y90-Y98.	Yes	Yes
**Ill-defined** (Chapter XVIII: Symptoms, signs and abnormal clinical and laboratory findings, not elsewhere classified)	R00-R09, R10-R19, R20-R23, R25-R29, R30-R39, R40-R46, R47-R49, R50-R69, R70-R79, R80-R82, R83-R89, R90-R94, R95-R99.	Yes	Yes
**Sensitivity analysis 1**			
**Tuberculosis**	A15-A19, B90, O98.0	No, because includes B90 - Sequelae of tuberculosis / O98.0 - Tuberculosis complicating pregnancy, childbirth and the puerperium	Yes
**Infection not tuberculosis**	A00-A09, A20-A28, A30-A49, A50-A64, A65-A69, A70-A74, A75-A79, A80-A89, A92-A99, B00-B09, B15-B19, B20-B24, B25-B34, B35-B49, B50-B64, B65-B83, B85-B89, B91-B94, B95-B98, B99, J09-J18.	No, because it divided Chapter I and included respiratory infections	Yes
**Respiratory not infections**	J00-J06, J20-J22, J30-J39, J40-J47, J60-J70, J80-J84, J85-J86, J90-J94, J95-J99.	No, because it excluded respiratory infections. Blocks respected.	Yes
**Ischaemic heart and Cerebrovascular diseases**	I20-I25, I60-I69.	No, because it selected only two blocks. Blocks structure respected.	Yes
**External causes or Ill-defined**	V01-V09, V10-V19, V20-V29, V30-V39, V40-V49, V50-V59, V60-V69, V70-V79, V80-V89, V90-V94, V95-V97, V98-V99, W00-W19, W20-W49, W50-W64, W65-W74, W75-W84, W85-W99, X00-X09, X10-X19, X20-X29, X30-X39, X40-X49, X50-X57, X58-X59, X60-X84, X85-Y09, Y10-Y34, Y35-Y36, Y40-Y59, Y60-Y69, Y70-Y82, Y83-Y84, Y85-Y89, Y90-Y98, R00-R09, R10-R19, R20-R23, R25-R29, R30-R39, R40-R46, R47-R49, R50-R69, R70-R79, R80-R82, R83-R89, R90-R94, R95-R99.	No, because merged two Chapters. Blocks structure respected.	Yes
**Sensitivity analysis 2**			
**Tuberculosis**	A15-A19, B90, O98 (underlying cause of death)	No, because includes B90 - Sequelae of tuberculosis / O98 - Tuberculosis complicating pregnancy, childbirth and the puerperium	Yes
**Tuberculosis related**	A15-A19, B90, O98 (mention in any line)	No, because includes B90 - Sequelae of tuberculosis / O98 - Tuberculosis complicating pregnancy, childbirth and the puerperium	No. Captures mention to any TB code in any line of death certificate.

TB – tuberculosis, WHO – world health organization

eFigure 1. Theoretical model used to support the research questions regarding vulnerability and TB

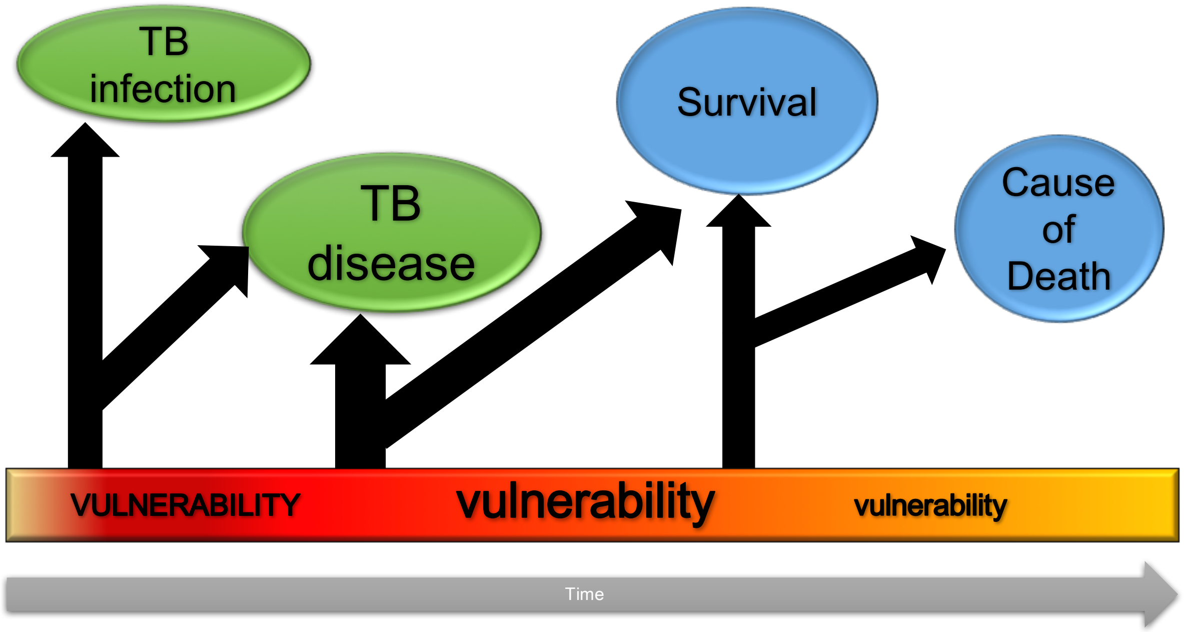

Vulnerable conditions and TB

Tuberculosis (TB) has been labelled a disease of poverty and health inequalities, and empirical data support the argument that social determinants are a cornerstone in the natural history of TB.[10] Although the increase in risk for active TB caused by some social factors is lower than some biological factors (e.g., HIV), social factors are more common in the population (e.g., poverty) and thus their population impact measures can be even higher than for HIV.[10-12]

We used a broad definition of vulnerability when starting this research program. It was defined as any situation in which exposed individuals are susceptible to physical, psychological, biological or socioeconomic stresses and have limited overall resources to cope with these stresses which thus have the potential to affect health. This broad concept of vulnerability and health was recently well-defined by a Delphi consensus, and was similar to that used for our theoretical model[13,14]. For TB, vulnerability means low socioeconomic position, lack of social capital, poor housing conditions, malnutrition, gender-associated vulnerability, and cultural barriers among others.[11] Vulnerable conditions increase markedly the risk of being infected, having latent TB infection (LTBI) and developing active disease (eFigure X).[15,16] Some vulnerable groups are usually more likely to have worse access to health care, poor adherence and outcomes.[17] Additionally, TB treatment is associated with high direct/indirect costs,[18,19] and a need for social support to achieves compliance.[20] Vulnerable groups face many of these problems.

Within this theoretical model, we hypothesised that patients with TB would have lower survival compared with the source population (both early during the 1-year of treatment and late up to 5-years), and that the survival would be even lower for those exposed to vulnerable conditions upon starting treatment. This rationale is illustrated in the eFigure 1, showing that the vulnerable conditions (or the circumstances that caused them before TB) are likely to be occurring dynamically over life, even after finishing TB treatment. Finally, we hypothesized that vulnerable conditions would be associated with cause-specific mortality, such as patients with social vulnerability dying more frequently due to external or ill-defined causes in the context of a LMIC. We selected six exposures frequently present in the TB literature as different constructs of vulnerability[21-23], labelling them social vulnerability (homeless, prison inmates)[10,22], health behaviours (alcohol and drug use)[12,21] and comorbidities (biological vulnerability), with diabetes and mental illness[21,24].

eFigure 2. Direct acyclic graph (DAG) for the association between different vulnerabilities (inmate, homelessness, alcohol and drug use, diabetes mellitus and mental disorder) and survival of newly-diagnosed tuberculosis patients.

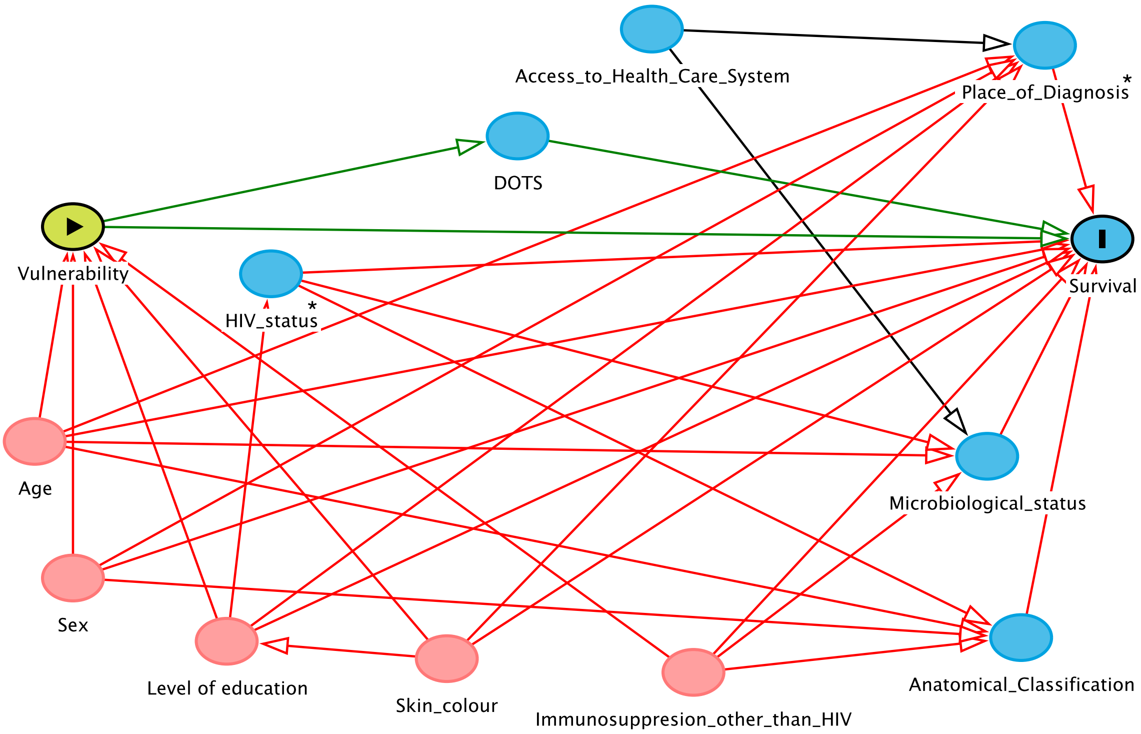

We built the Directed Acyclic Graph (DAG) based on the literature review and followed the DAG rules to define confounding factors, avoiding collider-stratification bias, and adjusting to close all back-door pathways [25,26]. *The relationship between HIV status, Place of diagnosis and Vulnerability were considered to have a circular/loop association, because the vulnerability can increase the risk of acquiring HIV, but the other way around (HIV increases the risk of vulnerability) is also possible; as well as for the variable access to the health care system, represented by Place of Diagnosis, when those with diabetes/mental disease could have an earlier diagnosis of TB, for instance [27,28]. Therefore, HIV status and Place of Diagnosis were adjusted for in the Cox models as strata, allowing different baseline hazards for each stratum[26]. We considered the offer of DOTS as a result of being vulnerable, thus acting as a mediator in the association between vulnerabilities and survival[2,29]. Finally, we adjusted for microbiological status and anatomical classification as surrogates of the pathogen-host interaction and risk factors for survival, aiming to improve precision of the estimates[4,21,26].

DAG built with the DAGitty tool (http://www.dagitty.net/).

Supplementary methods: Missing data / Multiple imputation

We had very few missing values for confounding variables, but for self-reported skin colour and education level (eTable 6A). To conduct the multiple imputation, we first investigated the patterns of missing variables. We assumed the missing values to be missing at random (MAR) and explored whether they were conditioned on observed variables, suggesting a MAR mechanism [30]. We conducted multiple imputation by fully conditional specification, therefore taking account for the non-linearity of our analysis model (ie, Cox model), using the command *smcfcs*.[31] We followed the recommended steps to build the imputed model, including all variables used in the final model as regular variables and the outcome, stratified by HIV status. eTable 6B specifies the method used for each imputed variable. We generated ten imputed datasets, fitted the final model to each of the imputed datasets, and combined the results using Rubin's rule[32]. The distribution of the imputed variables before-and-after the impution in on eTable 6C. As a sensitivity analysis for the multiple imputation, we conducted an analysis on a complete-case dataset.

eTable 6.A. Frequency of missing values in the five covariates who were imputed

Variable	Missing values in the whole cohort (n = 15501)
**Level of education, number of years of schooling**	**3200 (20.6%)**
**Self-reported skin colour**	**2658 (17.2%)**
**Microbiological status**	**1535 (9.9%)**
**Place of diagnosis**	**228 (1.5%)**
**Age**	**19 (0.1%)**

eTable 6.B. Methods used to impute the five covariates

Variable	Method used for imputation	Command
**Level of education**	**Ordered logistic regression**	**"ologit"**
**Self-reported skin colour**	**Multinomial logistic regression**	**"mlogit"**
**Microbiological status**	**Binary logistic regression**	**"logit"**
**Place of diagnosis**	**Multinomial logistic regression**	**"mlogit"**
**Age**	**Ordered logistic regression**	**"ologit"**

eTable 6.C. Frequency distribution of the five imputed covariates before and after imputation

Variable	Values	Whole cohort, Complete case dataset	Pooled final proportion of imputed variables[a]
**Level of education, number of years of schooling**	Illiterate	521 (4·2%)	4.4% (4.0-4.7%)
	1-3 years	1465 (11·9%)	12.1% (11.5-12.7%)
	4-7 years	4620 (37·6%)	37.7% (36.8-38.6%)
	8-11 years	4518 (36·7%)	36.3% (35.6-37.1%)
	12-14 years	798 (6·5%)	6.4% (6.0-6.9%)
	≥15 years	379 (3·1%)	3.0% (2.7-3.4%)
**Self-reported skin colour**	White	7129 (55·5%)	55.4% (54.4-56.3%)
	Brown/Mixed	3989 (31·1%)	31.0% (30.2-31.9%)
	Black	1488 (11·6%)	11.5% (10.9-12.1%)
	Asian/Indigenous	237 (1·9%)	2.1% (1.7-2.6%)
**Microbiological status**	Positive	10956 (78·5%)	75.6% (74.9-76.3%)
**Place of diagnosis**	Primary care/Outpatient	9349 (61·3%)	61.9% (61.1-62.6%)
	Emergency/Urgency facility	3300 (21·6%)	21.9% (21.2-22.5%)
	Hospitalized	2465 (16·1%)	16.3% (15.7-16.9%)
	Upon necropsy	159 (1·0%)	-
**Age**, years	15-25	2930 (18·9%)	19.1% (18.5-19.7%)
	25·1-35	4055 (26·2%)	26.3% (25.6-27.0%)
	35·1-45	3247 (21·0%)	21.1% (20.4-21.7%)
	45·1-55	2605 (16·8%)	16.7% (16.1-17.3%)
	55·1-65	1527 (9·9%)	9.8% (9.3-10.2%)
	>65·1	1118 (7·2%)	7.0% (6.6-7.5%)

[a] Pooled proportion from 10 imputed datasets using Rubin rules.

Supplementary methods: Description of sensitivity analysis

eTable 7. Description of sensitivity analyses

Sensitivity for	Reason
Grouping causes of death	This sensitivity analysis was necessary because the official ICD-10 divisions are important and standard, but have limitations for the current analysis, such as grouping pneumonia together with other non-infectious respiratory conditions. We therefore conducted two sensitivity analyses: A) re-grouping the underlying cause of death and B) considering TB as cause of death if mentioned as the underlying cause of death or if associated, in an exploratory analysis aiming to better capture the TB burden, particularly after TB-treatment (eg a patient who died of pneumonia, but with TB lung sequelae as associated cause of death).
TB population	Because of the importance of HIV and other immunosuppression status for cause of death, directly or indirectly according to the coding standardisation (ie, there are specific codes for cause of death in a patient with HIV, as well a patient with neoplasia), we ran the same main models on those patients who had no immunosuppression (HIV negative and without other immunossupression)
Modelling non-linear effect of diabetes mellitus (defined post-hoc)	We ran a flexible parametric survival modelling to explore the non-linearity of diabetes effect when modelled over 5-years because of its flexibility[33,34].
Multiple imputation (defined post-hoc)	We ran a sensitivity analysis on complete case dataset to evaluate the effect of multiple imputation
Starting point of the follow-up	We presented a sensitivity analysis choosing the starting point for the late mortality when the treatment outcome occurred, instead of the landmark analysis of 1-year after start of treatment.

eFigure3. Long-term survival of tuberculosis patients newly-diagnosed in 2010 in São Paulo State, Brazil.

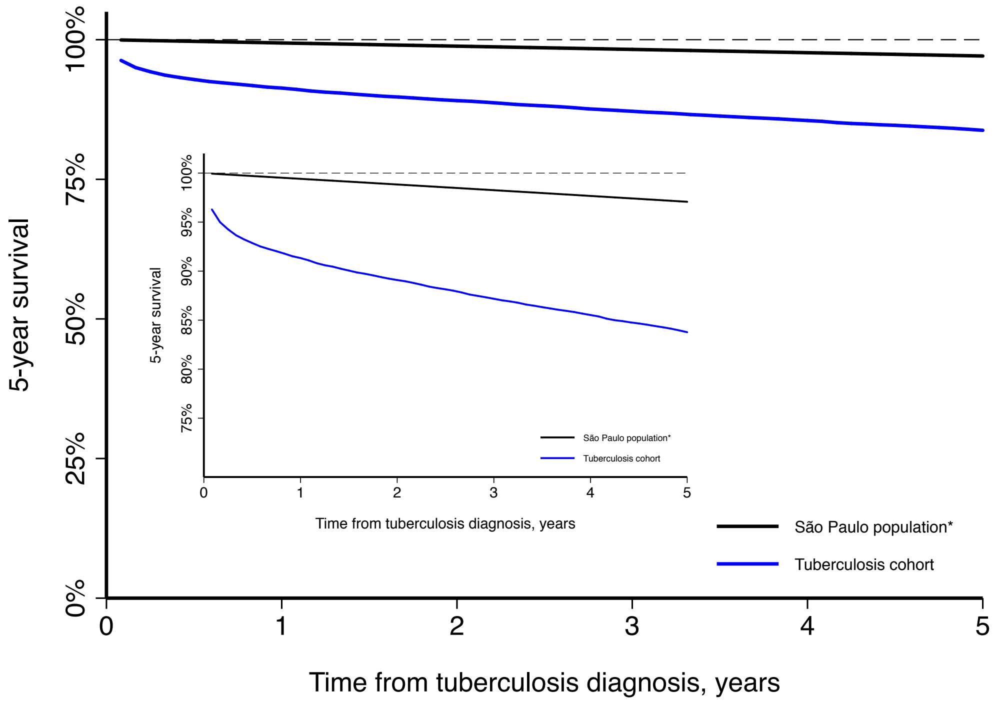

Number at risk (Tuberculosis cohort)

**15,342**	**14,011**	**13,667**	**13,374**	**13,120**	**7,233**

The inset shows the same data on a shorter range of survival probabilities in y-axis. * The source population was matched by age, sex and calendar year.

eTable 8. The crude mortality observed over 5-years of follow-up of newly-diagnosed tuberculosis cases stratified by characteristics at baseline and treatment outcome

Variable	Values	5-year mortality
**Age**, years[a]	15-25	118/2930 (4.0%)
	25.1-35	344/4055 (8.5%)
	35.1-45	577/3247 (17.8%)
	45.1-55	616/2605 (23.7%)
	55.1-65	456/1527 (29.9%)
	>65.1	539/1118 (48.2%)
**Sex**	Female	647/4683 (13.8%)
	Male	2013/10818 (18.6%)
**Self-reported skin colour** [b]	White	1236/7129 (17.3%)
	Brown/Mixed	590/3989 (14.8%)
	Black	282/1488 (19.0%)
	Asian/Indigenous	30/237 (12.7%)
**Level of education, number of years of schooling**[c]	Illiterate	146/521 (28.0%)
	1-3 years	291/1465 (19.9%)
	4-7 years	809/4620 (17.5%)
	8-11 years	469/4518 (10.4%)
	12-14 years	70/798 (8.8%)
	≥15 years	36/379 (9.5%)
**Inmate**	Yes	128/1609 (8.0%)
**Homelessness**	Yes	134/391 (34.3%)
**Alcohol use**	Yes	535/2053 (26.0%)
**Drug users**	Yes	184/1019 (18.1%)
**Diabetes mellitus**	Yes	232/880 (26.4%)
**Mental disorder**	Yes	85/336 (25.3%)
**HIV status**	Negative	1279/11155 (11.5%)
	Positive	729/1874 (38.9%)
	Unknown	652/2472 (26.4%)
**Immunosuppression from aetiologies other than HIV infection**	Yes	50/113 (44.3%)
**Anatomical classification**	PTB	2047/12458 (16.4%)
	PTB + EPTB	94/409 (23.0%)
	EPTB	357/2280 (15.7%)
	Miliary/Disseminated	162/354 (45.8%)
**Microbiological status** [d]	Positive	1660/10956 (15.2%)
**Place of diagnosis** [e]	Primary care/Outpatient	1133/9349 (12.1%)
	Emergency/Urgency facility	638/3300 (19.3%)
	Hospitalized	705/2465 (28.6%)
	Upon necropsy	-
**Treatment outcome**	Treatment success	1055/12227 (8.6%)
	Treatment failure	38/254 (15.0%)
	Death	-
	Loss to follow-up	289/1537 (18.8%)
	Not evaluated	31/236 (13.1%)

[a] Missing data: n = 19 (0.1%); [b] Missing data: n = 2658 (17.2%); [c] Missing data: n = 3200 (20.6%); [d] Missing data: n = 1535 (9.9%); [e] Missing data: n = 228 (1.5%).

PTB: pulmonary tuberculosis; EPTB: extrapulmonary tuberculosis

eFigure4. Cumulative hazard of mortality stratified by treatment outcome

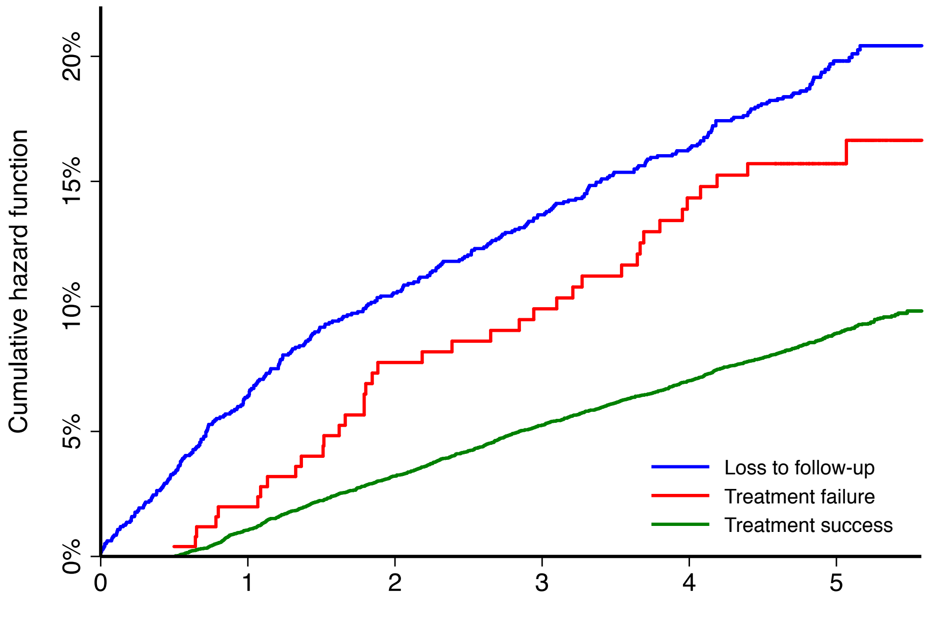

Number at risk (Tuberculosis cohort)

**Loss to follow-up^**	**1,773**	**1,659**	**1,592**	**1,543**	**1,503**	**842**
**Treatment failure**	**254**	**249**	**235**	**230**	**220**	**123**
**Treatment success**	**12,227**	**12,099**	**11,840**	**11,601**	**11,397**	**6,268**

^ For simplicity and illustration, the Loss to follow-up category also includes patients with outcome "Not evaluated". The cumulative hazard function was derived using the Nelson-Aalen estimator. The starting point was settled when the treatment outcome occurred.

eFigure 5. Causes of death of newly-diagnosed tuberculosis patients stratified by TB-HIV coinfection status and time of follow-up

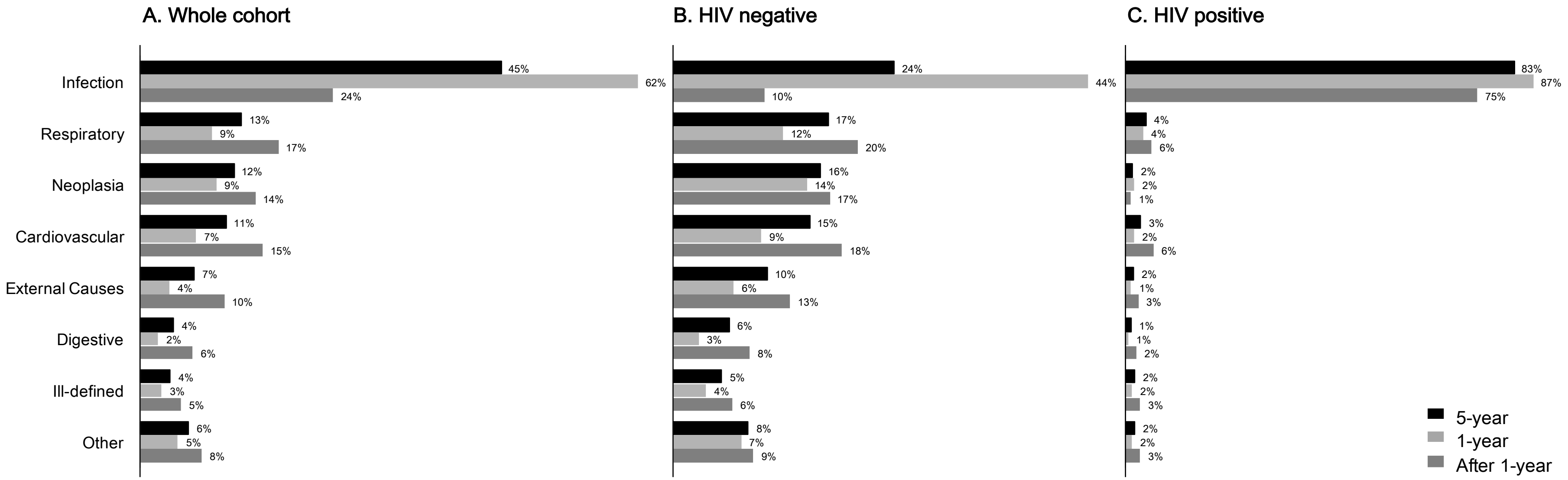

Classification based on the chapters defined by the standardized coding from the International Statistical Classification of Diseases and Related Health Problems 10th Revision (ICD-10). HIV – human immunodeficiency virus infection.

eFigure 6. Causes of death of newly-diagnosed tuberculosis cases, stratified by age and time from diagnosis.

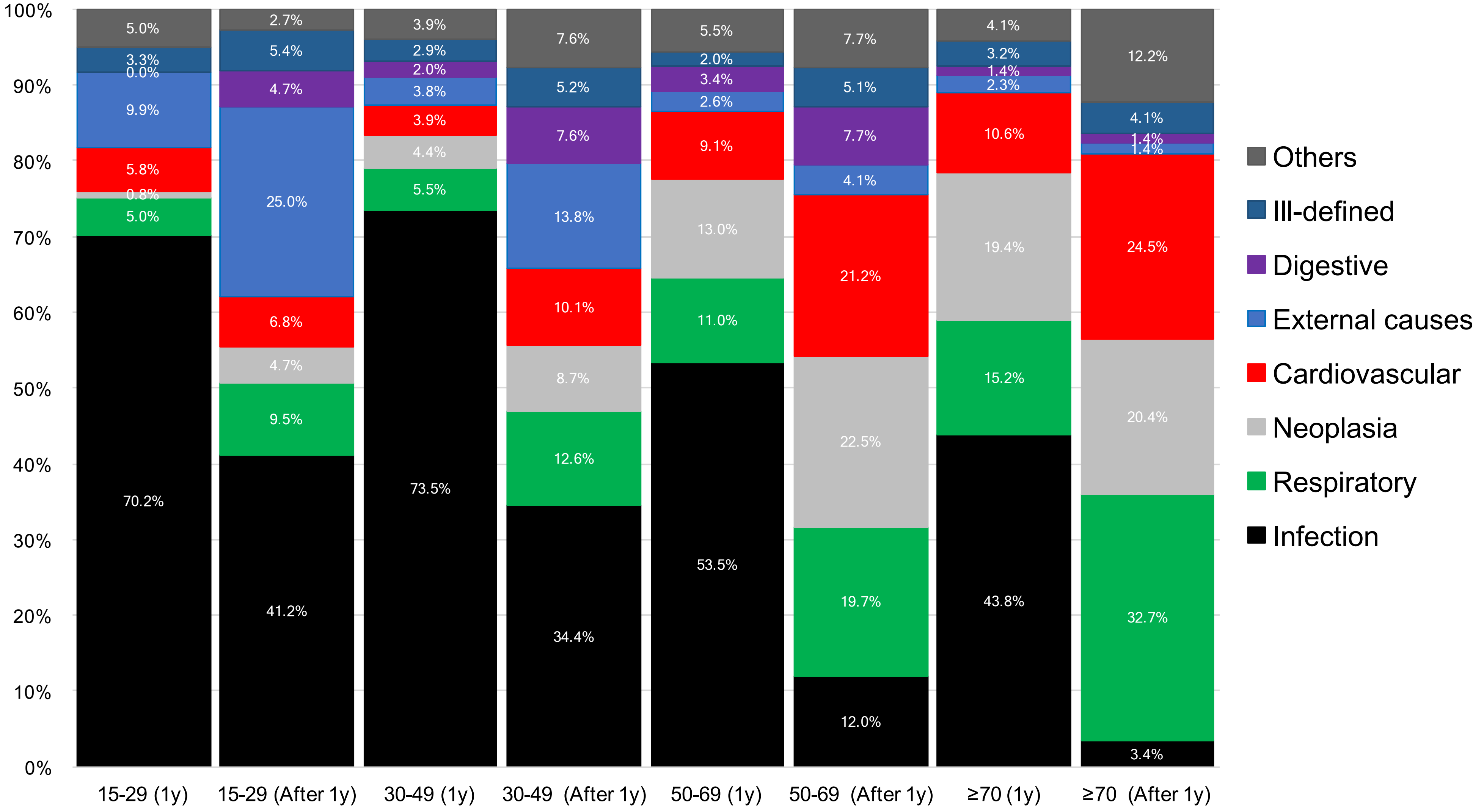

1y – deaths that occurred within the first year after diagnosis; After 1y – deaths that occurred after the first year of diagnosis

eTable 9. Top 10 causes of death during 5-year follow-up of tuberculosis patients newly diagnosed in 2010 in São Paulo State, Brazil.

**5-year**			**1-year**			**After 1-year**			**São Paulo State (2010-2015)**		
Rank	Cause	N (%)	Rank	Cause	N (%)	Rank	Cause	N (%)	Rank	Cause	N (%)
1	HIV disease	575 (22·1%)	1	Tuberculosis	460 (32·1%)	1	HIV disease	189 (16·1%)	1	Ischaemic heart disease	10·7%
2	Tuberculosis	520 (20·0%)	2	HIV disease	386 (26·9%)	2	Influenza and Pneumonia	104 (8·9%)	2	Cerebrovascular diseases	8·0%
3	Influenza and Pneumonia	168 (6·5%)	3	Influenza and Pneumonia	64 (4·5%)	3	Ischaemic heart disease	71 (6·1%)	3	Influenza and Pneumonia	7·2%
4	Chronic lower respiratory diseases	98 (3·8%)	4	Lung cancer	51 (3·6%)	4	Chronic lower respiratory diseases	67 (5·7%)	4	Gastrointestinal cancer	6·4%
5	Lung cancer	98 (3·8%)	5	Chronic lower respiratory diseases	31 (2·2%)	5	Tuberculosis	60 (5·1%)	5	Other forms of heart disease	5·4%
6	Ischaemic heart disease	97 (3·7%)	6	Ill-defined	30 (2·1%)	6	Liver diseases	53 (4·5%)	6	Ill-defined	4·5%
7	Ill-defined	81 (3·1%)	7	Ischaemic heart disease	26 (1·8%)	7	Ill-defined	51 (4·4%)	7	Chronic lower respiratory diseases	4·0%
8	Liver diseases	67 (2·6%)	8	Cerebrovascular diseases	25 (1·7%)	8	Gastrointestinal cancer	47 (4·0%)	8	Diabetes mellitus	3·4%
9	Gastrointestinal cancer	67 (2·6%)	9	Other forms of heart disease	21 (1·5%)	9	Lung cancer	47 (4·0%)	9	Hypertensive diseases	2·9%
10	Cerebrovascular diseases	58 (2·2%)	10	Gastrointestinal cancer	20 (1·4%)	10	Assault	41 (3·5%)	10	Lung cancer	2·9%

The first 10 causes in the 5-year, 1-year, after 1-year and São Paulo State correspond to 70%, 78%, 62% and 55% of deaths, respectively.

Classification based on the blocks defined by the standardized coding from the International Statistical Classification of Diseases and Related Health Problems 10th Revision (ICD-10). HIV – human immunodeficiency virus infection.

eTable 10. Top 10 causes of death during 5-year follow-up of newly diagnosed tuberculosis patients stratified by the exposures of interest.

Inmate			Homelessness			Alcohol use			Drug use			Diabetes mellitus			Mental disorder		
Rank	Cause	N (%)	Rank	Cause	N (%)	Rank	Cause	N (%)	Rank	Cause	N (%)	Rank	Cause	N (%)	Rank	Cause	N (%)
1	HIV disease	43 (34.4%)	1	Tuberculosis	36 (30.3%)	1	Tuberculosis	143 (27.2%)	1	HIV disease	73 (39.9%)	1	Tuberculosis	46 (19.9%)	1	Tuberculosis	21 (25.9%)
2	Assault	14 (11.2%)	2	HIV disease	27 (22.7%)	2	HIV disease	87 (16.5%)	2	Tuberculosis	33 (18.0%)	2	Diabetes mellitus	24 (10.4%)	2	Influenza and Pneumonia	10 (12.4%)
3	Tuberculosis	9 (7.2%)	3	Influenza and Pneumonia	16 (13.5%)	3	Influenza and Pneumonia	40 (7.6%)	3	Assault	13 (7.1%)	3	Ischaemic heart disease	22 (9.5%)	3	Chronic lower respiratory diseases	6 (7.4%)
4	Influenza and Pneumonia	8 (6.4%)	4	Other forms of heart disease	6 (5.0%)	4	Liver diseases	24 (4.6%)	4	Influenza and Pneumonia	10 (5.5%)	4	Influenza and Pneumonia	21 (9.1%)	4	HIV disease	6 (7.4%)
5	Intentional self-harm	6 (4.8%)	5	Ill-defined	5 (4.2%)	5	Ill-defined	24 (4.6%)	5	Liver diseases	5 (2.7%)	5	Gastrointestinal cancer	10 (4.3%)	5	Ill-defined	5 (6.2%)
6	Ischaemic heart disease	5 (4.0%)	6	Other diseases of the respiratory system	3 (2.5%)	6	Assault	16 (3.0%)	6	Chronic lower respiratory diseases	4 (2.2%)	6	Cerebrovascular diseases	8 (3.5%)	6	Ischaemic heart disease	4 (4.9%)
7	Liver diseases	4 (3.2%)	7	Cerebrovascular diseases	2 (1.7%)	7	Cerebrovascular diseases	16 (3.0%)	7	Event of undetermined intent	4 (2.2%)	7	Ill-defined	8 (3.5%)	7	Diabetes mellitus	3 (3.7%)
8	Event of undetermined intent	4 (3.2%)	8	Chronic lower respiratory diseases	2 (1.7%)	8	Ischaemic heart disease	14 (2.7%)	8	Ill-defined	4 (2.2%)	8	Hypertensive diseases	7 (3.0%)	8	Other forms of heart disease	3 (3.7%)
9	Ill-defined	4 (3.2%)	9	Falls	2 (1.7%)	9	Gastrointestinal cancer	13 (2.5%)	9	Ischaemic heart disease	4 (2.2%)	9	Lung cancer	7 (3.0%)	9	Liver diseases	2 (2.5%)
10	Cerebrovascular diseases	3 (2.4%)	10	Gastrointestinal cancer	2 (1.7%)	10	Lung cancer	12 (2.3%)	10	Cerebrovascular diseases	3 (1.6%)	10	Other forms of heart disease	7 (3.0%)	10	Cerebral palsy and other paralytic syndromes	1 (1.2%)

The first 10 causes of death for the exposures inmate, homelessness, alcohol use, drug use, diabetes mellitus and mental disorders correspond to 80%, 85%, 74%, 84%, 69%, and 75% of deaths, respectively.

Classification based on the blocks defined by the standardized coding from the International Statistical Classification of Diseases and Related Health Problems 10th Revision (ICD-10). HIV – human immunodeficiency virus infection.

eTable 11. Crude and age-sex adjusted hazard ratios for all-cause 5-year survival, 1-year survival and among those who survived the first year.

		Whole cohort, 5-year survival (n = 15,342)		Whole cohort, 1-year survival (n = 15,342)		Among those survived the first year (n = 14,011)	
**Exposures of interest**	**Values**	**All-cause mortality**		**All-cause mortality**		**All-cause mortality**	
		**Crude HR (95% CI), p-value**	**Age and sex adjusted HR (95% CI), p-value**	**Crude HR (95% CI), p-value**	**Age and sex adjusted HR (95% CI), p-value**	**Crude HR (95% CI), p-value**	**Age and sex adjusted HR (95% CI), p-value**
**Social vulnerability**							
Inmate	No	Reference	Reference	Reference	Reference	Reference	Reference
	Yes	0.43 (0.36-0.51), p<0.001	0.75 (0.62-0.90), p=0.002	0.37 (0.29-0.48), p < 0.001	0.68 (0.52-0.90), p=0.006	0.48 (0.38-0.62), p < 0.001	0.81 (0.63-1.05), p=0.104
Homelessness	No	Reference	Reference	Reference	Reference	Reference	Reference
	Yes	2.19 (1.82-2.63), p < 0.001	1.90 (1.58-2.29), p < 0.001	2.26 (1.77-2.89), p < 0.001	1.99 (1.56-2.55), p < 0.001	2.10 (1.58-2.78), p < 0.001	1.80 (1.35-2.38), p < 0.001
**Health behaviours**							
Alcohol use	No	Reference	Reference	Reference	Reference	Reference	Reference
	Yes	1.80 (1.63-1.98), p <0.001	1.44 (1.30-1.59), p <0.001	1.65 (1.44-1.89), p <0.001	1.35 (1.17-1.55), p <0.001	1.98 (1.72-2.27), p <0.001	1.55 (1.34-1.79), p <0.001
Drug use	No	Reference	Reference	Reference	Reference	Reference	Reference
	Yes	1.12 (0.96-1.30), p = 0.138	1.74 (1.49-2.03), p < 0.001	1.08 (0.88-1.33), p = 0.464	1.72 (1.39-2.13), p < 0.001	1.17 (0.94-1.45), p = 0.164	1.76 (1.40-2.20), p < 0.001
**Comorbidities**							
Diabetes mellitus	No	Reference	Reference	Reference	Reference	Reference	Reference
	Yes	1.32 (1.08-1.61), p = 0.007	0.76 (0.62-0.92), p = 0.006	1.34 (1.09-1.64), p = 0.006	0.75 (0.61-0.93), p = 0.008	2.12 (1.76-2.55), p < 0.001	1.21 (1.00-1.46), p = 0.053
	Time interaction[a]	1.17 (1.07-1.27), p < 0.001	1.16 (1.07-1.26), p = 0.001	-	-	-	-
Mental disorder	No	Reference	Reference	Reference	Reference	Reference	Reference
	Yes	1.57 (1.26-1.97), p < 0.001	1.30 (1.04-1.62), p = 0.022	1.74 (1.30-2.32), p < 0.001	1.43 (1.07-1.91), p = 0.016	1.38 (0.97-1.96), p = 0.073	1.14 (0.81-1.63), p = 0.452
**Combined**							
Alcohol or drug use or homelessness	No	Reference	Reference	Reference	Reference	Reference	Reference
	Yes	1.66 (1.52-1.82), p<0.001	1.54 (1.40-1.69), p<0.001	1.54 (1.36-1.75), p<0.001	1.45 (1.28-1.65), p<0.001	1.80 (1.58-2.05), p<0.001	1.63 (1.43-1.87), p<0.001
Alcohol and drug use and homelessness	1 factor	1.61 (1.46-1.78)	1.42 (1.28-1.57)	1.48 (1.29-1.70)	1.33 (1.15-1.53)	1.77 (1.54-2.04)	1.53 (1.32-1.77)
	2 factors	1.80 (1.51-2.15)	2.08 (1.73-2.48)	1.69 (1.32-2.16)	2.00 (1.55-2.55)	1.93 (1.50-2.50)	2.17 (1.67-2.82)
	3 factors	2.29 (1.42-3.69)	2.53 (1.56-4.08)	2.64 (1.46-4.78)	2.97 (1.63-5.39)	1.83 (0.82-4.09)	1.97 (0.88-4.41)

[a] Time-varying effect of diabetes mellitus interacting with survival time. CI – confidence interval; HR – hazard ratio

eTable 12. The association between social vulnerability, health behaviours, and comorbidities and 5-year survival of newly-diagnosed tuberculosis patients in fully adjusted models, fitting a Cox proportional hazard model with time-dependent effect for diabetes mellitus and a flexible parametric survival model.

		Whole cohort, 5-year survival (n = 15342)	
**Exposures of interest**	**Values**	**All-cause mortality**	
		**Cox proportional hazard model HR (95% CI), p-value**	**Flexible Parametric Survival[a] HR (95% CI), p-value**
**Social vulnerability**			
Inmate	No	Reference	Reference
	Yes	0.88 (0.73-1.07), p=0.189	0.88 (0.73-1.07), p=0.201
Homelessness	No	Reference	Reference
	Yes	1.51 (1.25-1.83), p < 0.001	1.53 (1.26-1.85), p < 0.001
**Health behaviours**			
Alcohol use	No	Reference	Reference
	Yes	1.36 (1.22-1.51), p <0.001	1.36 (1.22-1.52), p <0.001
Drug use	No	Reference	Reference
	Yes	1.20 (1.02-1.41), p = 0.030	1.20 (1.02-1.41), p = 0.030
**Comorbidities**			
Diabetes mellitus	No	Reference	
	Yes	0.83 (0.67-1.02), p = 0.067	
	Time interaction	1.15 (1.05-1.25), p = 0.001	
Mental disorder	No	Reference	Reference
	Yes	1.09 (0.87-1.38), p = 0.439	1.08 (0.86-1.35), p = 0.527
**Combined vulnerabilities**			
Alcohol or drug use or homelessness	No	Reference	Reference
	Yes	1.45 (1.32-1.60), p<0.001	1.45 (1.32-1.60), p<0.001
Alcohol and drug use and homelessness	1 factor	1.38 (1.25-1.54)	1.38 (1.24-1.53)
	2 factors	1.73 (1.44-2.08)	1.76 (1.46-2.11)
	3 factors	2.11 (1.30-3.42)	2.13 (1.31-3.44)

[a] The flexible parametric survival model was fit with restricted cubic splines, with 3 degrees of freedom for the baseline hazard function and 2 degrees of freedom for the time-dependent effect (diabetes mellitus). CI – confidence interval; HR – hazard ratio.

eFigure 7. Fully adjusted hazard ratio for diabetes mellitus from the parametric flexible survival model over 5-years from diagnosis.

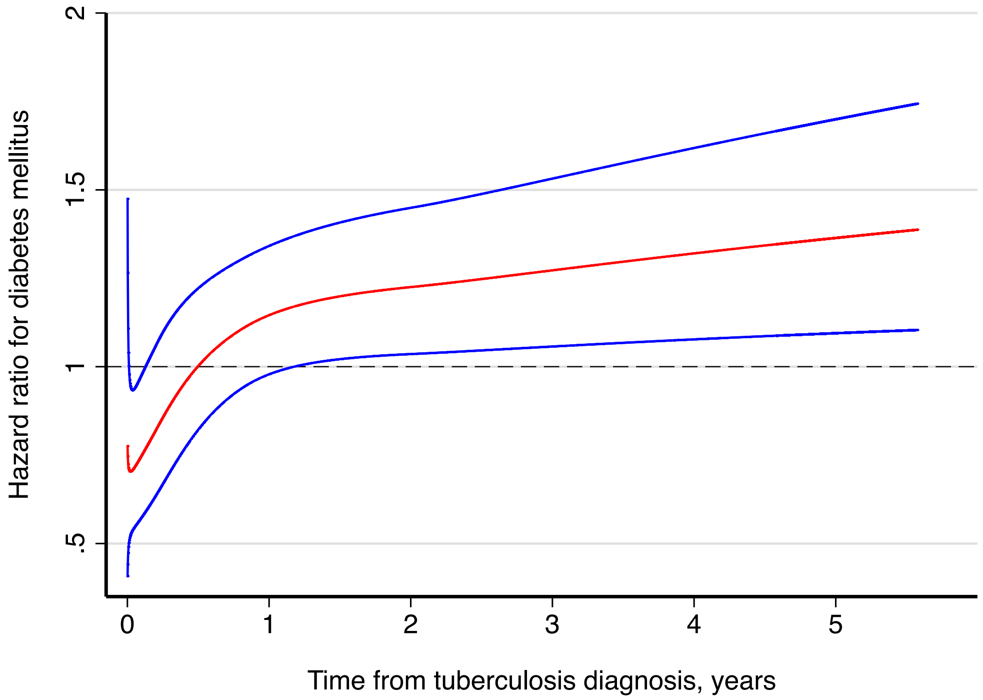

eTable 13. Sensitivity analysis for fully adjusted models for all-cause survival while using the multiple imputation or complete case analysis

		5-year survival (Whole cohort)		1-year survival (Whole cohort)		Among those who survived the first year	
		Multiple imputed (n = 15342)	Complete case analysis (n = 9699)	Multiple imputed (n = 15342)	Complete case analysis (n = 9699)	Multiple imputed (n = 14011)	Complete case analysis (n = 9052)
Exposures of interest	Values	Adj. HR (95% CI), p-value[a]	Adj. HR (95% CI), p-value	Adj. HR (95% CI), p-value[a]	Adj. HR (95% CI), p-value	Adj. HR (95% CI), p-value[a]	Adj. HR (95% CI), p-value
**Social vulnerability**							
Inmate	No	Reference	Reference	Reference	Reference	Reference	Reference
	Yes	0·88 (0·73-1·07), p=0·189	0·90 (0·71-1·15), p=0·411	0·88 (0·67-1·17), p = 0·388	0·87 (0·59-1·28), p = 0·475	0·86 (0·66-1·12), p = 0·261	0·91 (0·67-1·25), p = 0·575
Homelessness	No	Reference	Reference	Reference	Reference	Reference	Reference
	Yes	1·51 (1·25-1·83), p < 0·001	1·52 (1·16-1·99), p = 0·002	1·54 (1·19-1·98), p = 0·001	1·57 (1·07-2·31), p = 0·023	1·48 (1·11-1·97), p = 0·008	1·49 (1·04-2·15), p = 0·031
**Health behaviours**							
Alcohol use	No	Reference	Reference	Reference	Reference	Reference	Reference
	Yes	1·36 (1·22-1·51), p <0·001	1·27 (1·10-1·47), p = 0·001	1·30 (1·12-1·52), p = 0·001	1·23 (0·99-1·52), p = 0·063	1·42 (1·22-1·66), p < 0·001	1·32 (1·09-1·60), p = 0·005
Drug use	No	Reference	Reference	Reference	Reference	Reference	Reference
	Yes	1·20 (1·02-1·41), p = 0·030	1·32 (1·08-1·62), p = 0·007	1·13 (0·90-1·41), p = 0·302	1·30 (0·96-1·75), p = 0·087	1·31 (1·03-1·65), p = 0·026	1·35 (1·03-1·78), p = 0·033
**Comorbidities**							
Diabetes mellitus	No	Reference	Reference	Reference	Reference	Reference	Reference
	Yes	0·83 (0·67-1·02), p = 0·067	0·75 (0·56-1·01), p = 0·055	0·83 (0·67-1·03), p = 0·084	0·76 (0·56-1·03), p = 0·074	1·28 (1·06-1·56), p = 0·011	1·25 (0·98-1·60), p = 0·067
Time interaction[b]		1·15 (1·05-1·25), p = 0·001	1·17 (1·05-1·31), p = 0·005				
Mental disorder	No	Reference	Reference	Reference	Reference	Reference	Reference
	Yes	1·09 (0·87-1·38), p = 0·439	1·05 (0·75-1·46), p = 0·795	1·25 (0·93-1·68), p = 0·147	1·37 (0·90-2·09), p = 0·147	0·92 (0·64-1·32), p = 0·649	0·74 (0·44-1·27), p = 0·275
**Combined**							
Alcohol or drug use or homelessness	No	Reference	Reference	Reference	Reference	Reference	Reference
	Yes	1·45 (1·32-1·60), p<0·001	1·43 (1·26-1·62), p<0·001	1·37 (1·20-1·57), p<0·001	1·33 (1·10-1·61), p = 0·003	1·55 (1·35-1·78), p<0·001	1·51 (1·28-1·79), p<0·001
Alcohol and drug use and homelessness	1	1·38 (1·25-1·54)	1·34 (1·17-1·54)	1·31 (1·13-1·51)	1·23 (1·00-1·52)	1·47 (1·27-1·71)	1·44 (1·20-1·73)
	2	1·73 (1·44-2·08)	1·77 (1·41-2·24)	1·57 (1·22-2·02)	1·67 (1·18-2·35)	1·97 (1·51-2·57)	1·90 (1·39-2·61)
	3	2·11 (1·30-3·42)	1·99 (1·03-3·86)	2·36 (1·29-4·30)	2·90 (1·29-6·55)	1·75 (0·77-3·93)	1·21 (0·39-3·78)

[a] Fully adjusted models in 10 mulitple imputed datasets, [b] Time-varying effect of diabetes mellitus interacting with survival time.

eTable 14. Sensitivity analysis for the association between vulnerable conditions and all-cause mortality considering starting the follow-up time after 1 year or after treatment definition occurrence

		Starting at 1-year (n = 14,011)			Starting after treatment definition occurrence (n = 14,254)		
**Exposures of interest**	**Values**	**Patients (n)**	**Deaths (n)**	**Adj. HR (95% CI), p-value[a]**	**Patients (n)**	**Deaths (n)**	**Adj. HR (95% CI), p-value[a]**
**Social vulnerability**							
Inmate	No	12462	1102 (8·8%)	Reference	12697	1337 (10·5%)	Reference
	Yes	1549	68 (4·4%)	0·86 (0·66-1·12), p = 0·261	1557	76 (4·9%)	0.80 (0.63-1.03), p=0.080
Homelessness	No	13703	1119 (8·2%)	Reference	13931	1347 (9·7%)	Reference
	Yes	308	51 (16·6%)	1·48 (1·11-1·97), p = 0·008	323	66 (20·4%)	1.53 (1.18-1.97), p=0.001
**Health behaviours**							
Alcohol use	No	12241	918 (7·5%)	Reference	12446	1123 (9·0%)	Reference
	Yes	1770	252 (14·2%)	1·42 (1·22-1·66), p < 0·001	1808	290 (16·0%)	1.30 (1.13-1.50), p < 0·001
Drug use	No	13088	1082 (8·3%)	Reference	13304	1298 (9·8%)	Reference
	Yes	923	88 (9·5%)	1·31 (1·03-1·65), p = 0·026	950	115 (12·1%)	1.46 (1.19-1.80), p<0.001
**Comorbidities**							
Diabetes mellitus	No	13239	1046 (7·9%)	Reference	13464	1271 (9·4%)	Reference
	Yes	772	124 (16·1%)	1·28 (1·06-1·56), p = 0·011	790	142 (18·0%)	1.21 (1.01-1.45), p=0.039
Mental disorder	No	13728	1138 (8·3%)	Reference	13958	1368 (9·8%)	Reference
	Yes	283	32 (11·3%)	0·92 (0·64-1·32), p = 0·649	296	45 (15·2%)	1.08 (0.79-1.46), p=0.633
**Combined**							
Alcohol or drug use or homelessness	No	11560	854 (7·4%)	Reference	11743	1037 (8·8%)	Reference
	Yes	2451	316 (12·9%)	1·55 (1·35-1·78), p<0·001	2511	376 (15·0%)	1.52 (1.34-1.72), p<0·001
Alcohol and drug use and homelessness	1 factor	1946	247 (12·7%)	1·47 (1·27-1·71)	1988	289 (14·5%)	1.42 (1.24-1.63)
	2 factors	460	63 (13·7%)	1·97 (1·51-2·57)	476	79 (16·6%)	1.98 (1.56-2.51)
	3 factors	45	6 (13·3%)	1·75 (0·77-3·93)	47	8 (17·0%)	1.86 (0.92-3.76)

[a] Fully adjusted models in 10 multiple imputed datasets.

eFigure 8. Cumulative incidence function for 5 and 1-year cause-specific mortality stratified by TB-HIV coinfection status.

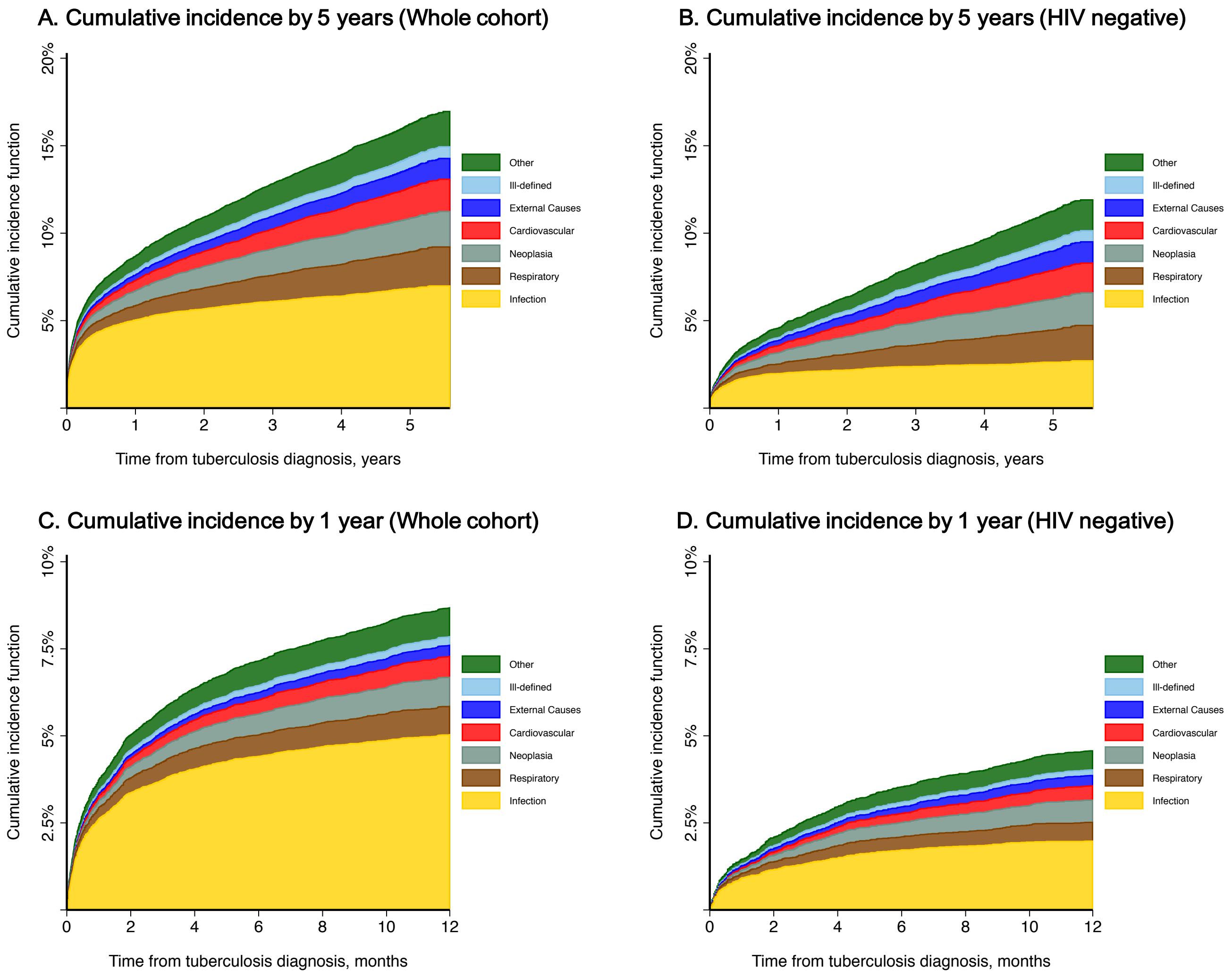

Classification based on the chapters defined by the standardized coding from the International Statistical Classification of Diseases and Related Health Problems 10th Revision (ICD-10). HIV – human immunodeficiency virus infection.

eTable 15. The association between social vulnerability, health behaviours, and comorbidities and 5-year cause-specific mortality of tuberculosis patients newly-diagnosed in 2010 in São Paulo State, Brazil (Population: Whole cohort).

		5-year cause-specific mortality (n = 15342)					
**Exposures of interest**	**Values**	**Infection**	**Respiratory**	**Neoplasia**	**Cardiovascular**	**External**	**Ill-defined**
		**Adj. cHR (95% CI)[a], p-value**	**Adj. cHR (95% CI)[a], p-value**	**Adj. cHR (95% CI)[a], p-value**	**Adj. cHR (95% CI)[a], p-value**	**Adj. cHR (95% CI)[a], p-value**	**Adj. cHR (95% CI)[a], p-value**
**Social vulnerability**							
Inmate	Yes	0.83 (0.62-1.11), p=0.213	0.76 (0.39-1.48), p=0.421	0.61 (0.24-1.52), p=0.288	1.16 (0.60-2.22), p=0.664	1.40 (0.90-2.20), p=0.140	0.79 (0.30-2.08), p=0.631
Homelessness	Yes	1.49 (1.14-1.96), p=0.004	2.36 (1.48-3.74), p<0.001	0.58 (0.21-1.56), p=0.280	1.59 (0.83-3.06), p=0.161	0.63 (0.23-1.72), p=0.363	1.73 (0.69-4.37), p=0.246
**Health behaviours**							
Alcohol use	Yes	1.55 (1.31-1.83), p<0.001	1.03 (0.75-1.40), p=0.860	0.84 (0.59-1.20), p=0.347	1.24 (0.89-1.75), p=0.206	1.51 (1.03-2.23), p=0.037	2.34 (1.43-3.81), p=0.001
Drug use	Yes	1.22 (0.98-1.51), p=0.081	1.35 (0.80-2.28), p=0.262	1.21 (0.55-2.64), p=0.636	1.31 (0.70-2.43), p=0.397	1.46 (0.90-2.38), p=0.122	0.53 (0.19-1.52), p=0.240
**Comorbidities**							
Diabetes mellitus	Yes	0.84 (0.64-1.11), p=0.212	0.88 (0.60-1.27), p=0.481	0.60 (0.40-0.91), p=0.017	1.70 (1.23-2.35), p=0.001	0.89 (0.43-1.85), p=0.757	1.43 (0.74-2.73), p=0.285
Mental disorder	Yes	0.82 (0.55-1.23), p=0.339	1.76 (1.09-2.85), p=0.021	0.36 (0.12-1.14), p=0.082	1.00 (0.49-2.05), p=0.994	0.87 (0.27-2.78), p=0.813	2.39 (1.00-5.67), p=0.049
**Alcohol or drug use or homelessness**	Yes	1.60 (1.39-1.85), p<0.001	1.23 (0.93-1.62), p=0.139	0.83 (0.59-1.15), p=0.256	1.43 (1.06-1.95), p=0.021	1.66 (1.18-2.33), p=0.004	2.06 (1.31-3.25), p=0.002

[a] Fully adjusted models in 10 mulitple imputed datasets. Adj. cHR – adjusted cause-specific hazard ratios; CI – confidence interval.

eTable 16. The association between social vulnerability, health behaviours, and comorbidities and 5-year cause-specific mortality of tuberculosis patients newly diagnosed in 2010 in São Paulo State, Brazil (Population: Not immunocompromised cohort).

		5-year cause-specific survival (n = 11061)					
**Exposures of interest**	**Values**	**Infection**	**Respiratory**	**Neoplasia**	**Cardiovascular**	**External**	**Ill-defined**
		**Adj. cHR (95% CI)[a], p-value**	**Adj. cHR (95% CI)[a], p-value**	**Adj. cHR (95% CI)[a], p-value**	**Adj. cHR (95% CI)[a], p-value**	**Adj. cHR (95% CI)[a], p-value**	**Adj. cHR (95% CI)[a], p-value**
**Social vulnerability**							
Inmate	Yes	0.43 (0.15-1.17), p=0.099	0.43 (0.13-1.41), p=0.164	0.54 (0.17-1.76), p=0.307	0.97 (0.41-2.31), p=0.940	1.37 (0.79-2.36), p=0.263	0.77 (0.22-2.67), p=0.678
Homelessness	Yes	2.15 (1.33-3.50), p=0.002	3.29 (1.96-5.52), p<0.001	0.44 (0.11-1.80), p=0.257	0.69 (0.22-2.20), p=0.533	0.68 (0.21-2.17), p=0.514	2.25 (0.79-6.41), p=0.127
**Health behaviours**							
Alcohol use	Yes	1.98 (1.50-2.63), p=0.000	1.05 (0.73-1.51), p=0.777	0.94 (0.62-1.43), p=0.784	1.29 (0.87-1.91), p=0.202	1.36 (0.88-2.10), p=0.169	2.20 (1.23-3.95), p=0.008
Drug use	Yes	1.37 (0.86-2.17), p=0.184	1.23 (0.61-2.49), p=0.567	0.95 (0.34-2.65), p=0.920	1.71 (0.84-3.50), p=0.142	1.72 (1.00-2.96), p=0.049	0.70 (0.21-2.35), p=0.561
**Comorbidities**							
Diabetes mellitus	Yes	0.93 (0.62-1.40), p=0.727	0.86 (0.53-1.37), p=0.515	0.80 (0.50-1.30), p=0.378	1.79 (1.23-2.62), p=0.003	0.99 (0.45-2.16), p=0.982	1.60 (0.77-3.34), p=0.205
Mental disorder	Yes	1.16 (0.62-2.17), p=0.637	1.51 (0.81-2.81), p=0.199	0.52 (0.16-1.65), p=0.267	1.18 (0.51-2.70), p=0.698	0.69 (0.16-2.87), p=0.609	2.84 (1.08-7.47), p=0.034
**Alcohol or drug use or homelessness**	Yes	2.22 (1.71-2.89), p<0.001	1.31 (0.94-1.82), p=0.111	0.85 (0.57-1.26), p=0.414	1.43 (1.00-2.05), p=0.053	1.58 (1.07-2.33), p=0.021	2.19 (1.27-3.79), p=0.005

[a] Fully adjusted models in 10 mulitple imputed datasets. Adj. cHR – adjusted cause-specific hazard ratios; CI – confidence interval.

eFigure 9. Fully adjusted cause-specific hazard ratios for 5-year cause-specific mortality (Population: Not immunocompromised cohort)

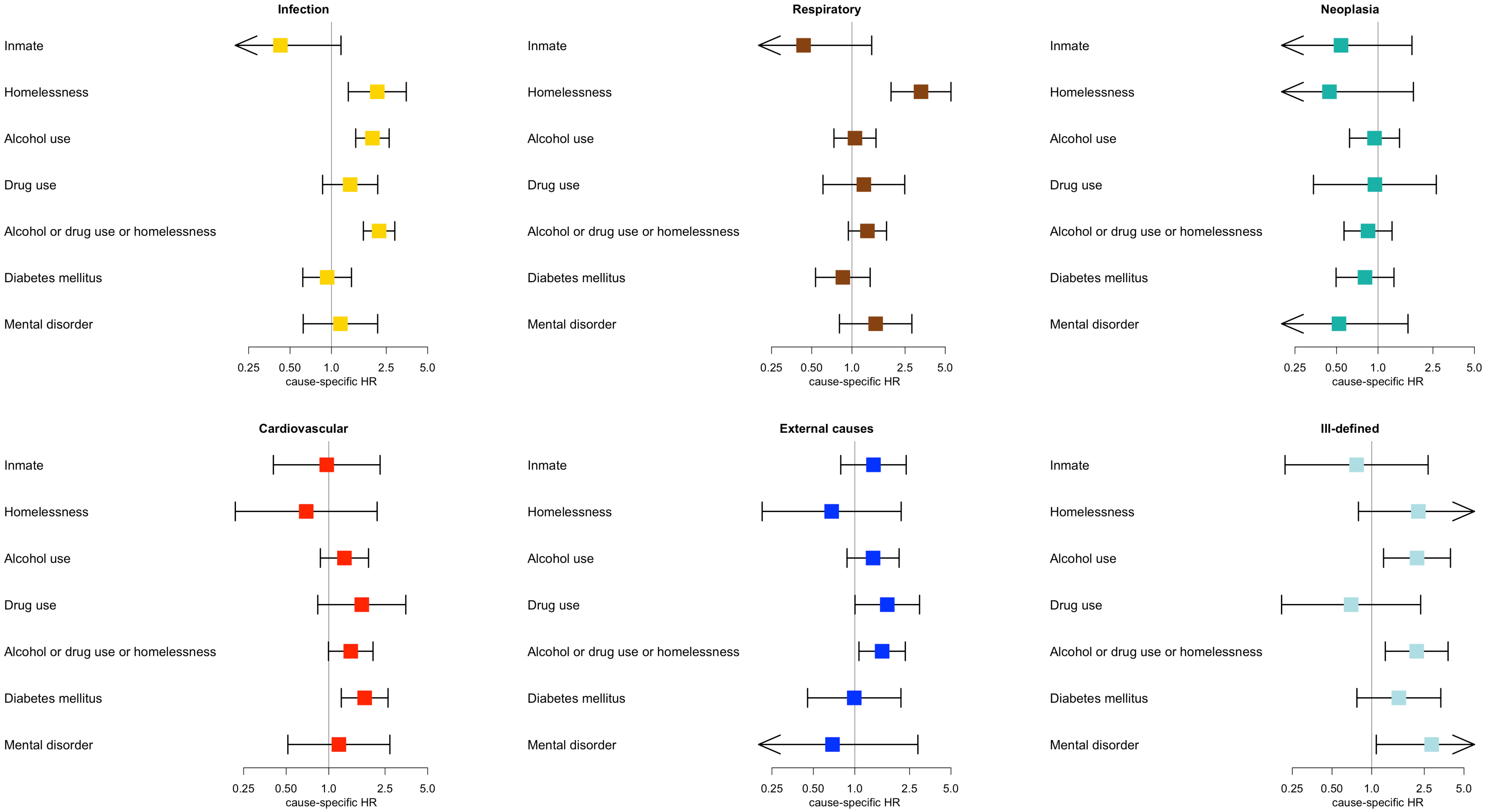

Adjusted cause-specific hazard ratios are shown as boxes and 95% confidence intervals as horizontal lines. The arrow represents the direction of the confidence interval value, truncated at the horizontal axes limit.
HR – hazard ratio

eTable 17. The association between social vulnerability, health behaviours, and comorbidities and 5-year cause-specific mortality among those patients who survived the first year in 2010 in São Paulo State, Brazil.

		Cause-specific mortality among those patients alive after the 1-year (n = 14,011)					
**Exposures of interest**	**Values**	**Infection**	**Respiratory**	**Neoplasia**	**Cardiovascular**	**External**	**Ill-defined**
		**Adj. cHR (95% CI)[a], p-value**	**Adj. cHR (95% CI)[a], p-value**	**Adj. cHR (95% CI)[a], p-value**	**Adj. cHR (95% CI)[a], p-value**	**Adj. cHR (95% CI)[a], p-value**	**Adj. cHR (95% CI)[a], p-value**
**Social vulnerability**							
Inmate	Yes	0.86 (0.54-1.39), p=0.543	0.66 (0.28-1.56), p=0.346	0.67 (0.24-1.91), p=0.456	0.95 (0.42-2.14), p=0.899	1.64 (0.98-2.74), p=0.059	0.21 (0.03-1.60), p=0.133
Homelessness	Yes	1.70 (1.01-2.84), p=0.045	2.67 (1.54-4.63), p<0.001	0.44 (0.11-1.81), p=0.258	2.04 (0.98-4.25), p=0.056	0.69 (0.21-2.21), p=0.530	1.69 (0.51-5.55), p=0.389
**Health behaviours**							
Alcohol use	Yes	1.36 (0.96-1.92), p=0.086	1.23 (0.85-1.78), p=0.270	0.99 (0.64-1.52), p=0.957	1.44 (0.97-2.14), p=0.069	1.51 (0.95-2.41), p=0.081	2.15 (1.16-3.99), p=0.015
Drug use	Yes	1.66 (1.13-2.44), p=0.009	1.44 (0.77-2.70), p=0.256	0.96 (0.34-2.67), p=0.932	1.00 (0.45-2.23), p=0.997	1.49 (0.84-2.63), p=0.169	1.04 (0.35-3.09), p=0.94
**Comorbidities**							
Diabetes mellitus	Yes	1.00 (0.55-1.82), p=0.993	1.01 (0.64-1.59), p=0.979	0.80 (0.49-1.32), p=0.384	2.21 (1.51-3.23), p<0.001	0.87 (0.35-2.17), p=0.761	1.03 (0.40-2.63), p=0.955
Mental disorder	Yes	0.37 (0.12-1.17), p=0.090	1.66 (0.88-3.11), p=0.116	0.43 (0.10-1.73), p=0.232	0.55 (0.17-1.75), p=0.312	0.83 (0.20-3.45), p=0.794	1.28 (0.30-5.46), p=0.737
**Alcohol or drug use or homelessness**	Yes	1.75 (1.32-2.31), p<0.001	1.48 (1.06-2.07), p=0.022	0.84 (0.55-1.28), p=0.416	1.65 (1.15-2.37), p=0.006	1.67 (1.11-2.50), p=0.013	2.41 (1.37-4.26), p=0.002

[a] Fully adjusted models in 10 mulitple imputed datasets. Adj. cHR – adjusted cause-specific hazard ratios; CI – confidence interval.

eFigure 10. Fully adjusted cause-specific hazard ratios for 5-year cause-specific mortality (Population: Among those who survived the first year)

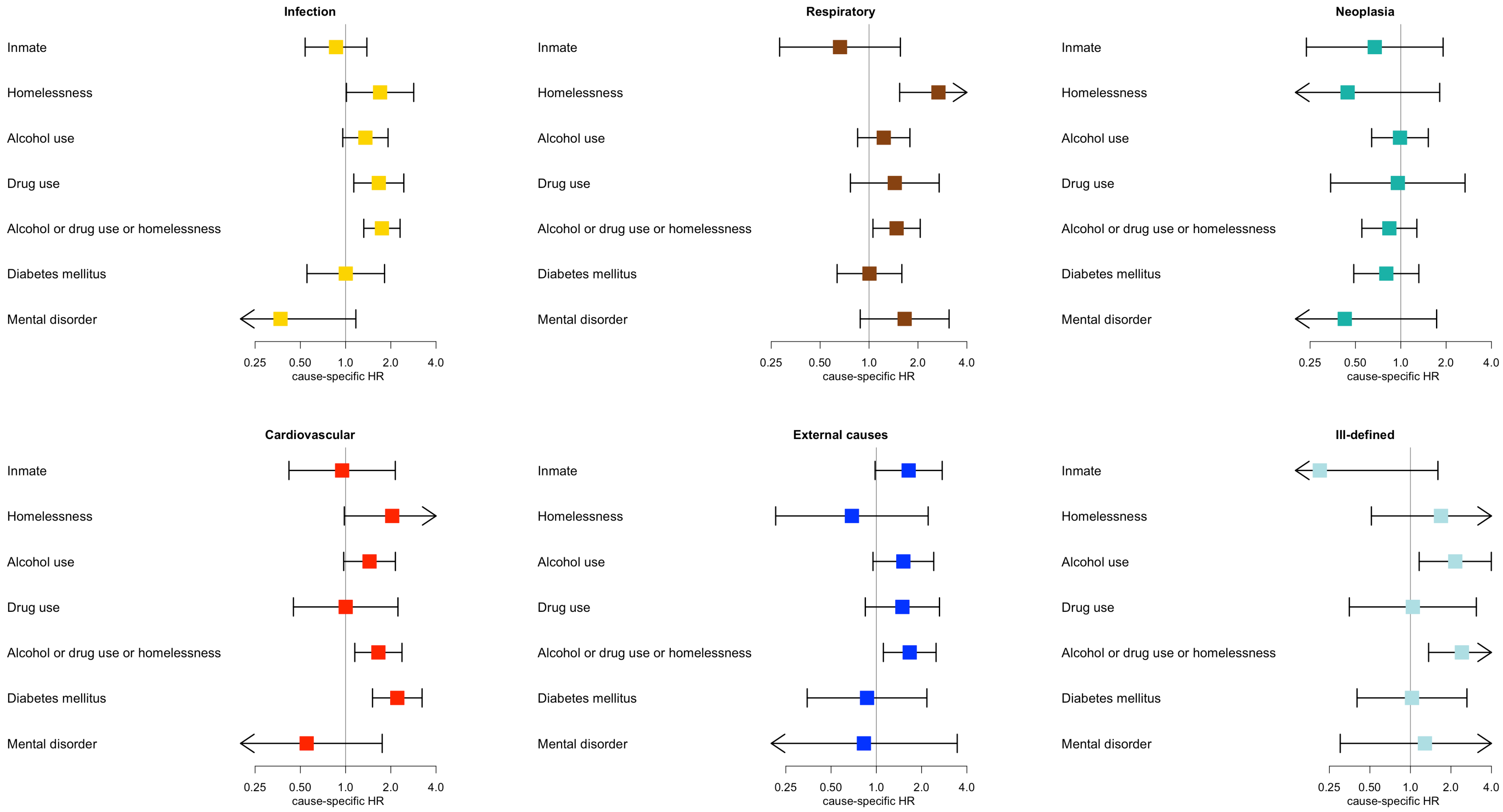

Adjusted cause-specific hazard ratios are shown as boxes and 95% confidence intervals as horizontal lines. The arrow represents the direction of the confidence interval value, truncated at the horizontal axes limit.
HR – hazard ratio

eFigure 11. Cumulative incidence function for 5 and 1-year cause-specific mortality (sensitivity analysis for cause of death)

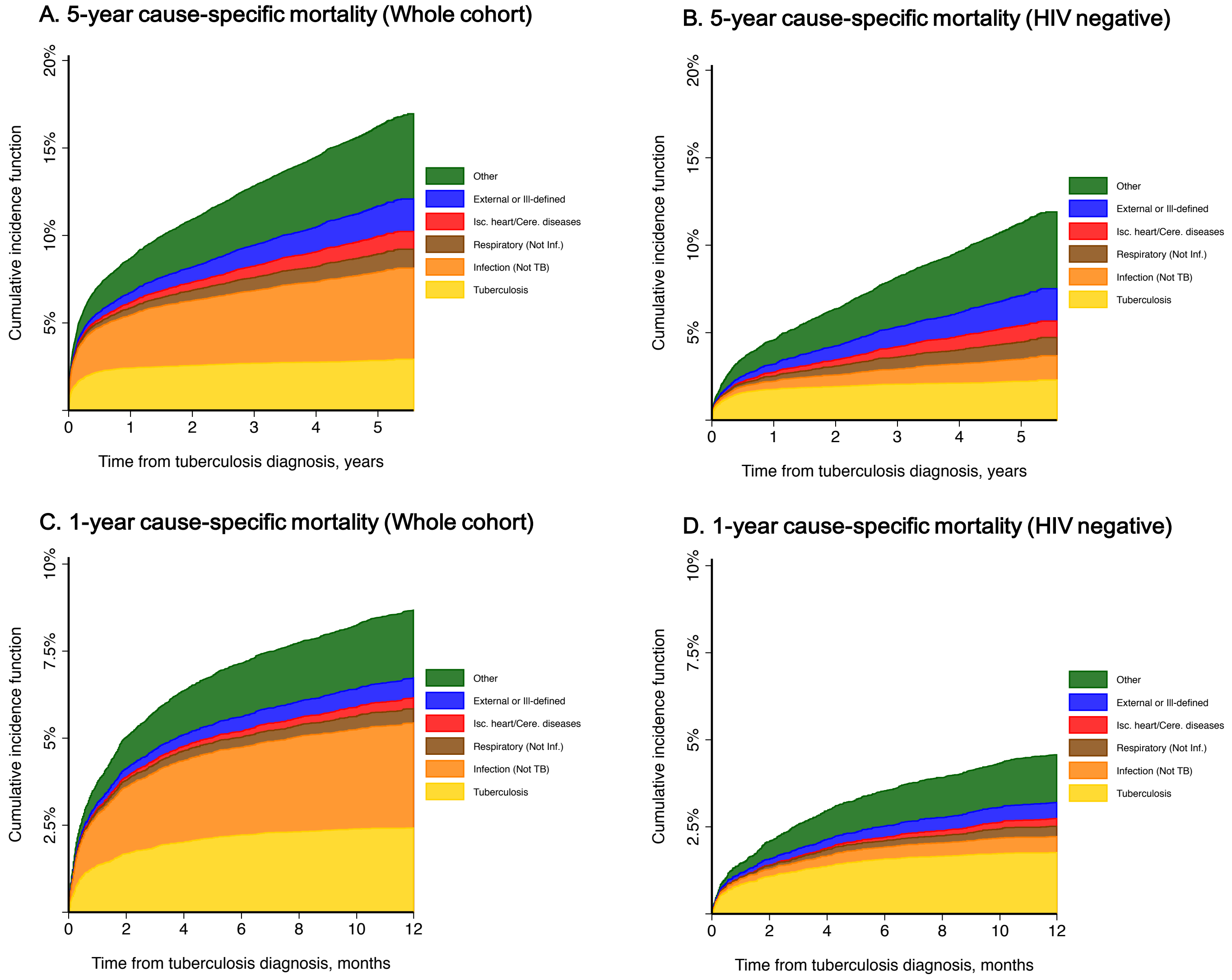

eTable 18. The association between social vulnerability, health behaviours, and comorbidities and 5-year cause-specific mortality of tuberculosis patients newly diagnosed in 2010 in São Paulo State, Brazil (sensitivity analysis for cause of death).

		5-year cause-specific survival (n = 15342)				
**Exposures of interest**	**Values**	**Tuberculosis**	**Infection (Not-Tuberculosis)**	**Respiratory (Not pneumonia)**	**Ischaemic heart or Cerebrovascular diseases**	**External or ill-defined**
		**Adj. cHR (95% CI)[a], p-value**	**Adj. cHR (95% CI)[a], p-value**	**Adj. cHR (95% CI)[a], p-value**	**Adj. cHR (95% CI)[a], p-value**	**Adj. cHR (95% CI)[a], p-value**
**Social vulnerability**						
Inmate	Yes	0.49 (0.24-1.00), p=0.050	0.97 (0.72-1.31), p=0.853	0.40 (0.09-1.68), p=0.209	1.63 (0.74-3.58), p=0.228	1.29 (0.87-1.93), p=0.208
Homelessness	Yes	2.14 (1.47-3.12), p<0.001	1.44 (1.04-1.97), p=0.027	1.28 (0.51-3.20), p=0.602	0.86 (0.27-2.75), p=0.799	0.97 (0.49-1.90), p=0.919
**Health behaviours**						
Alcohol use	Yes	1.86 (1.47-2.35), p<0.001	1.31 (1.06-1.62), p=0.011	0.67 (0.40-1.12), p=0.127	1.36 (0.87-2.11), p=0.177	1.77 (1.30-2.39), p<0.001
Drug use	Yes	1.30 (0.88-1.92), p=0.193	1.22 (0.95-1.57), p=0.110	2.25 (0.99-5.11), p=0.052	1.41 (0.63-3.18), p=0.406	1.17 (0.75-1.80), p=0.488
**Comorbidities**						
Diabetes mellitus	Yes	0.87 (0.63-1.20), p=0.393	0.98 (0.69-1.38), p=0.889	0.55 (0.30-0.99), p=0.048	1.89 (1.24-2.86), p=0.003	1.13 (0.70-1.82), p=0.627
Mental disorder	Yes	1.20 (0.74-1.94), p=0.469	0.86 (0.52-1.41), p=0.549	1.72 (0.86-3.45), p=0.126	0.89 (0.32-2.46), p=0.818	1.48 (0.75-2.94), p=0.259
**Alcohol or drug use or homelessness**	Yes	2.06 (1.66-2.56), p<0.001	1.38 (1.16-1.63), p<0.001	0.90 (0.58-1.40), p=0.635	1.54 (1.03-2.31), p=0.035	1.80 (1.37-2.36), p<0.001

[a] Fully adjusted models in 10 mulitple imputed datasets. Adj. cHR – adjusted cause-specific hazard ratios; CI – confidence interval.

eTable 19. The association between social vulnerability, health behaviours, and comorbidities and 5-year cause-specific mortality of tuberculosis patients newly diagnosed in 2010 in São Paulo State, Brazil (sensitivity analysis for causes of death. Population: Not immunocompromised cohort).

		5-year cause-specific survival (n = 11061)				
**Exposures of interest**	**Values**	**Tuberculosis**	**Infection (Not-Tuberculosis)**	**Respiratory (Not pneumonia)**	**Ischaemic heart or Cerebrovascular diseases**	**External or ill-defined**
		**Adj. cHR (95% CI)[a], p-value**	**Adj. cHR (95% CI)[a], p-value**	**Adj. cHR (95% CI)[a], p-value**	**Adj. cHR (95% CI)[a], p-value**	**Adj. cHR (95% CI)[a], p-value**
**Social vulnerability**						
Inmate	Yes	0.48 (0.17-1.32), p=0.154	0.41 (0.10-1.72), p=0.223	0.35 (0.05-2.63), p=0.310	1.92 (0.76-4.84), p=0.166	1.26 (0.77-2.07), p=0.354
Homelessness	Yes	2.21 (1.32-3.70), p=0.002	3.67 (2.06-6.57), p<0.001	2.09 (0.83-5.26), p=0.117	0.41 (0.06-2.98), p=0.377	1.13 (0.52-2.45), p=0.755
**Health behaviours**						
Alcohol use	Yes	2.06 (1.53-2.78), p<0.001	1.51 (1.00-2.27), p=0.048	0.66 (0.37-1.21), p=0.179	1.45 (0.88-2.39), p=0.147	1.61 (1.13-2.28), p=0.008
Drug use	Yes	1.18 (0.70-1.97), p=0.537	1.48 (0.76-2.88), p=0.251	1.40 (0.42-4.65), p=0.582	1.72 (0.66-4.50), p=0.265	1.43 (0.88-2.33), p=0.154
**Comorbidities**						
Diabetes mellitus	Yes	0.85 (0.54-1.35), p=0.503	1.28 (0.77-2.13), p=0.346	0.58 (0.28-1.20), p=0.141	2.14 (1.32-3.46), p=0.002	1.25 (0.74-2.12), p=0.412
Mental disorder	Yes	1.25 (0.65-2.41), p=0.510	1.18 (0.51-2.73), p=0.699	1.65 (0.71-3.85), p=0.244	1.11 (0.35-3.60), p=0.856	1.48 (0.68-3.23), p=0.327
**Alcohol or drug use or homelessness**	Yes	2.30 (1.73-3.04), p<0.001	1.84 (1.26-2.68), p=0.002	0.83 (0.49-1.41), p=0.483	1.57 (0.98-2.50), p=0.059	1.78 (1.29-2.43), p<0.001

[a] Fully adjusted models in 10 mulitple imputed datasets. Adj. cHR – adjusted cause-specific hazard ratios; CI – confidence interval.

eFigure 12. Fully adjusted cause-specific hazard ratios for 5-year cause-specific mortality (sensitivity analysis for causes of death)

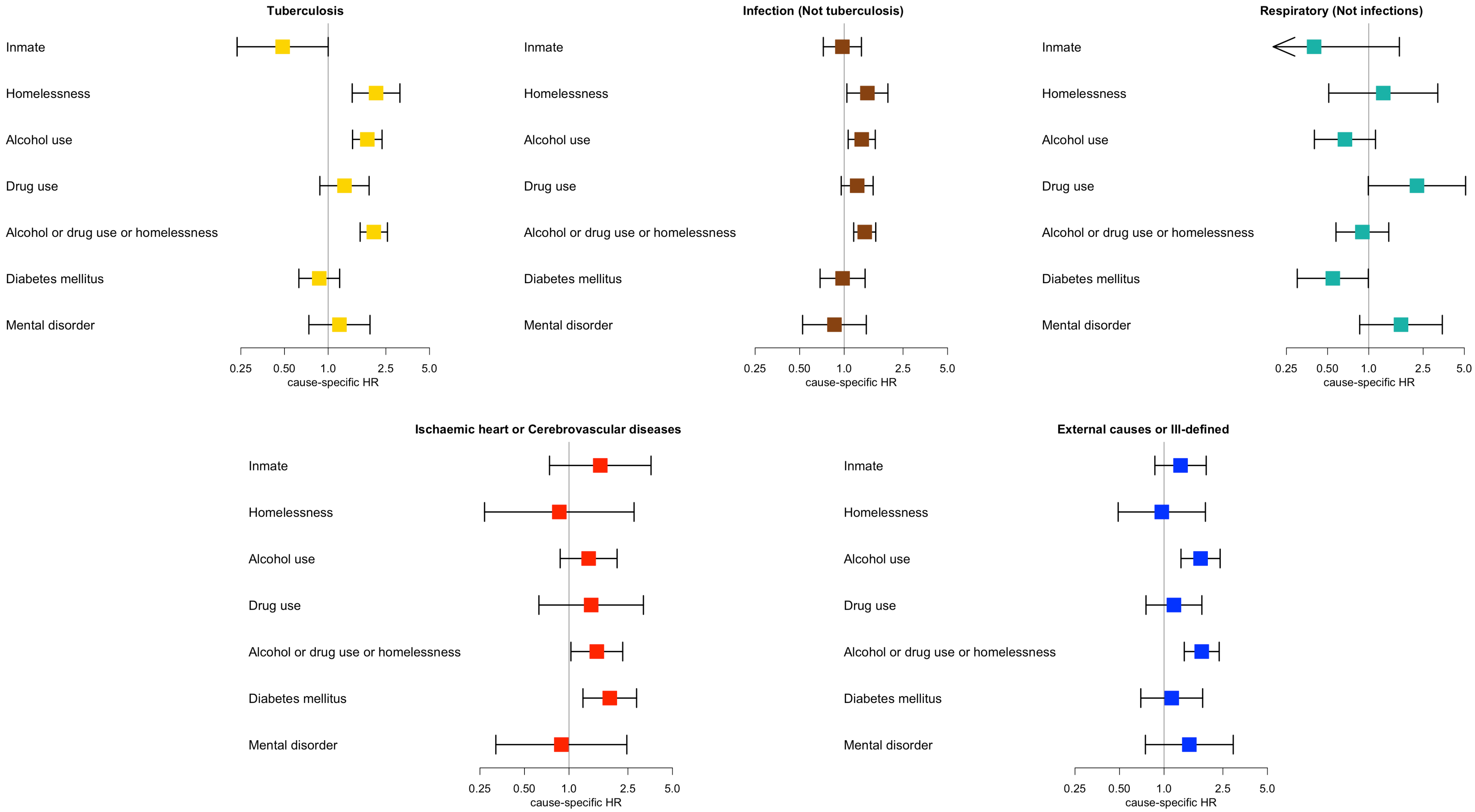

Adjusted cause-specific hazard ratios are shown as boxes and 95% confidence intervals as horizontal lines. The arrow represents the direction of the confidence interval value, truncated at the horizontal axes limit.
HR – hazard ratio

eFigure 13. Fully adjusted cause-specific hazard ratios for 5-year cause-specific mortality (sensitivity analysis for causes of death. Population: Not immunocompromised cohort)

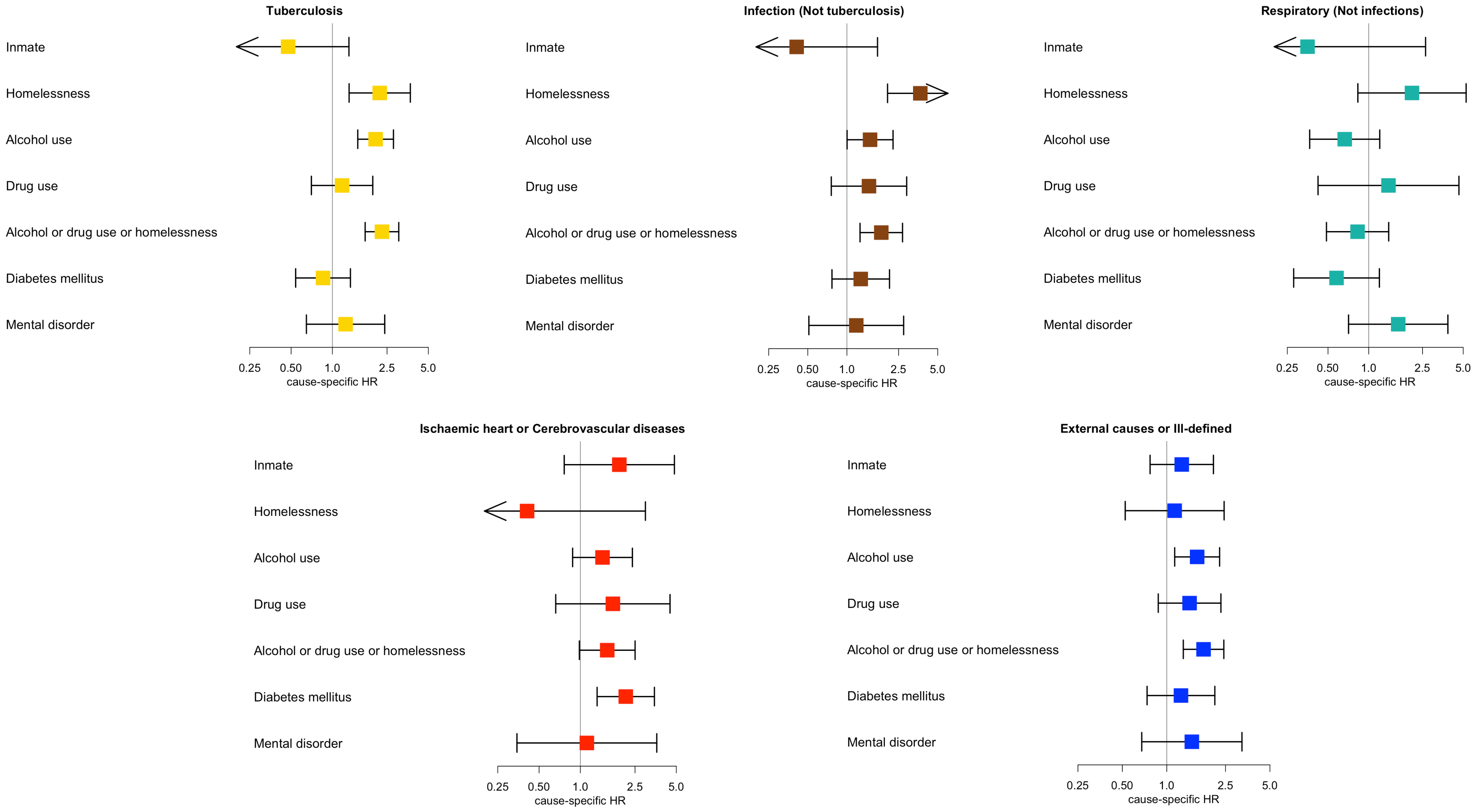

Adjusted cause-specific hazard ratios are shown as boxes and 95% confidence intervals as horizontal lines. The arrow represents the direction of the confidence interval value, truncated at the horizontal axes limit.
HR – hazard ratio

eTable 20. Fully adjusted cause-specific hazard ratios for 5-year cause-specific mortality among those patients who survived the first year in 2010 in São Paulo State, Brazil (sensitivity analysis for cause of death)

		Cause-specific mortality among those patients alive after the 1-year (n = 14,011)				
**Exposures of interest**	**Values**	**Tuberculosis**	**Infection (Not-Tuberculosis)**	**Respiratory (Not pneumonia)**	**Ischaemic heart or Cerebrovascular diseases**	**External or ill-defined**
		**Adj. cHR (95% CI)[a], p-value**	**Adj. cHR (95% CI)[a], p-value**	**Adj. cHR (95% CI)[a], p-value**	**Adj. cHR (95% CI)[a], p-value**	**Adj. cHR (95% CI)[a], p-value**
**Social vulnerability**						
Inmate	Yes	0.36 (0.08-1.53), p=0.164	0.94 (0.60-1.48), p=0.799	0.61 (0.14-2.66), p=0.515	1.58 (0.63-3.98), p=0.330	1.31 (0.81-2.10), p=0.266
Homelessness	Yes	3.84 (1.75-8.42), p=0.001	1.96 (1.23-3.12), p=0.005	1.28 (0.39-4.17), p=0.679	0.91 (0.22-3.75), p=0.893	0.98 (0.43-2.26), p=0.966
**Health behaviours**						
Alcohol use	Yes	1.17 (0.63-2.20), p=0.617	1.56 (1.14-2.13), p=0.006	0.82 (0.44-1.54), p=0.535	1.49 (0.89-2.49), p=0.128	1.70 (1.17-2.47), p=0.005
Drug use	Yes	1.50 (0.67-3.36), p=0.330	1.42 (0.97-2.08), p=0.067	2.06 (0.70-6.05), p=0.189	1.4 (0.53-3.66), p=0.496	1.37 (0.83-2.26), p=0.221
**Comorbidities**						
Diabetes mellitus	Yes	0.69 (0.25-1.94), p=0.485	1.43 (0.91-2.25), p=0.120	0.61 (0.29-1.28), p=0.191	2.37 (1.47-3.84), p<0.001	0.93 (0.48-1.79), p=0.831
Mental disorder	Yes	0.48 (0.06-3.61), p=0.479	0.84 (0.40-1.73), p=0.626	1.58 (0.62-3.99), p=0.336	0.32 (0.04-2.32), p=0.258	1.01 (0.37-2.78), p=0.988
**Alcohol or drug use or homelessness**	Yes	1.82 (1.06-3.12), p=0.030	1.77 (1.36-2.29), p<0.001	1.11 (0.65-1.92), p=0.697	1.68 (1.05-2.70), p=0.030	1.89 (1.36-2.62), p<0.001

[a] Fully adjusted models in 10 mulitple imputed datasets. Adj. cHR – adjusted cause-specific hazard ratios; CI – confidence interval.

eFigure 14. Fully adjusted cause-specific hazard ratios for 5-year cause-specific mortality among those patients who survived the first year in 2010 in São Paulo State, Brazil (sensitivity analysis for cause of death)

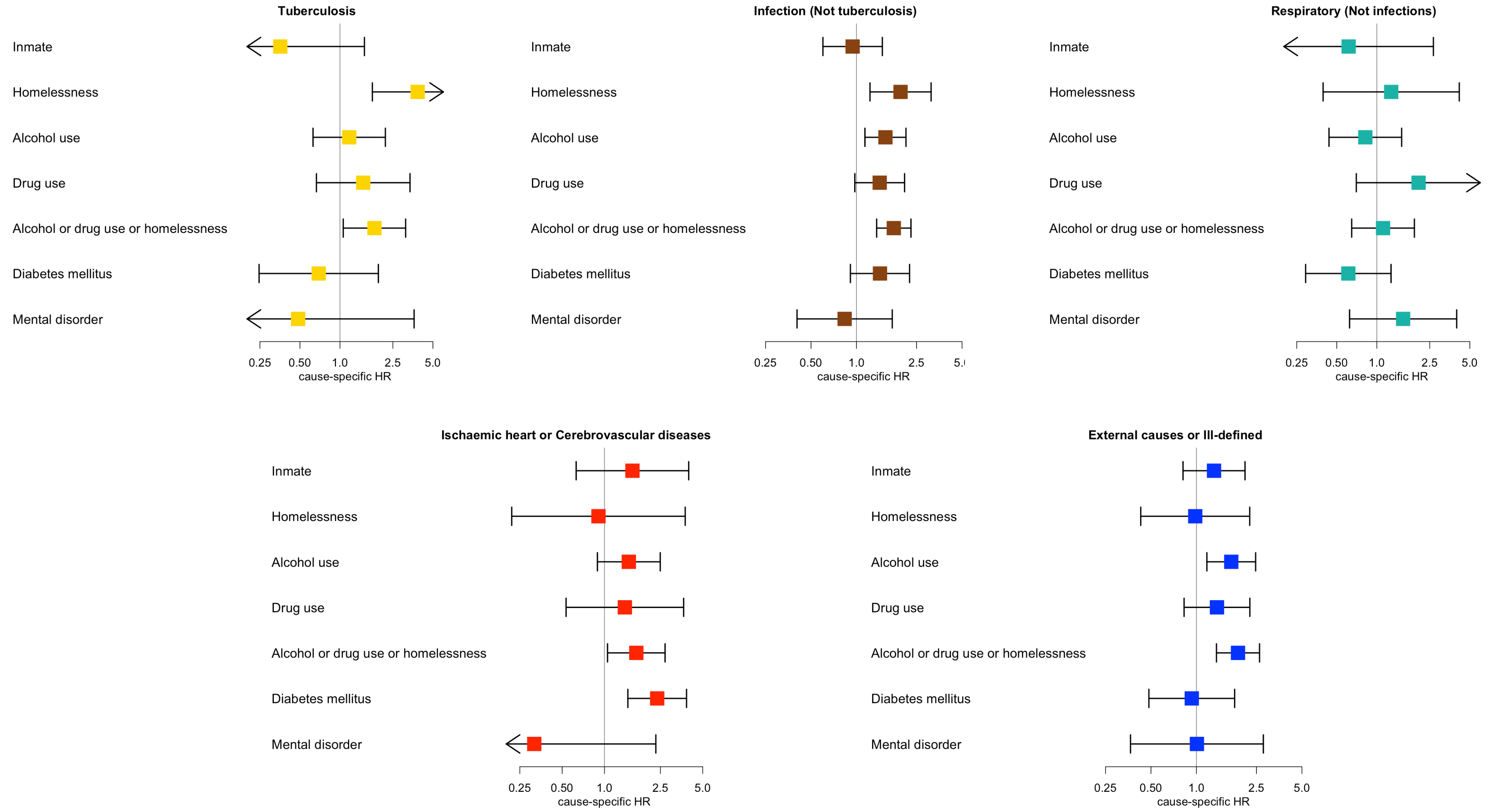

Adjusted cause-specific hazard ratios are shown as boxes and 95% confidence intervals as horizontal lines. The arrow represents the direction of the confidence interval value, truncated at the horizontal axes limit.
HR – hazard ratio

eFigure 15. Cumulative incidence function for 5 and 1-year TB related cause-specific mortality stratified by TB-HIV coinfection status.

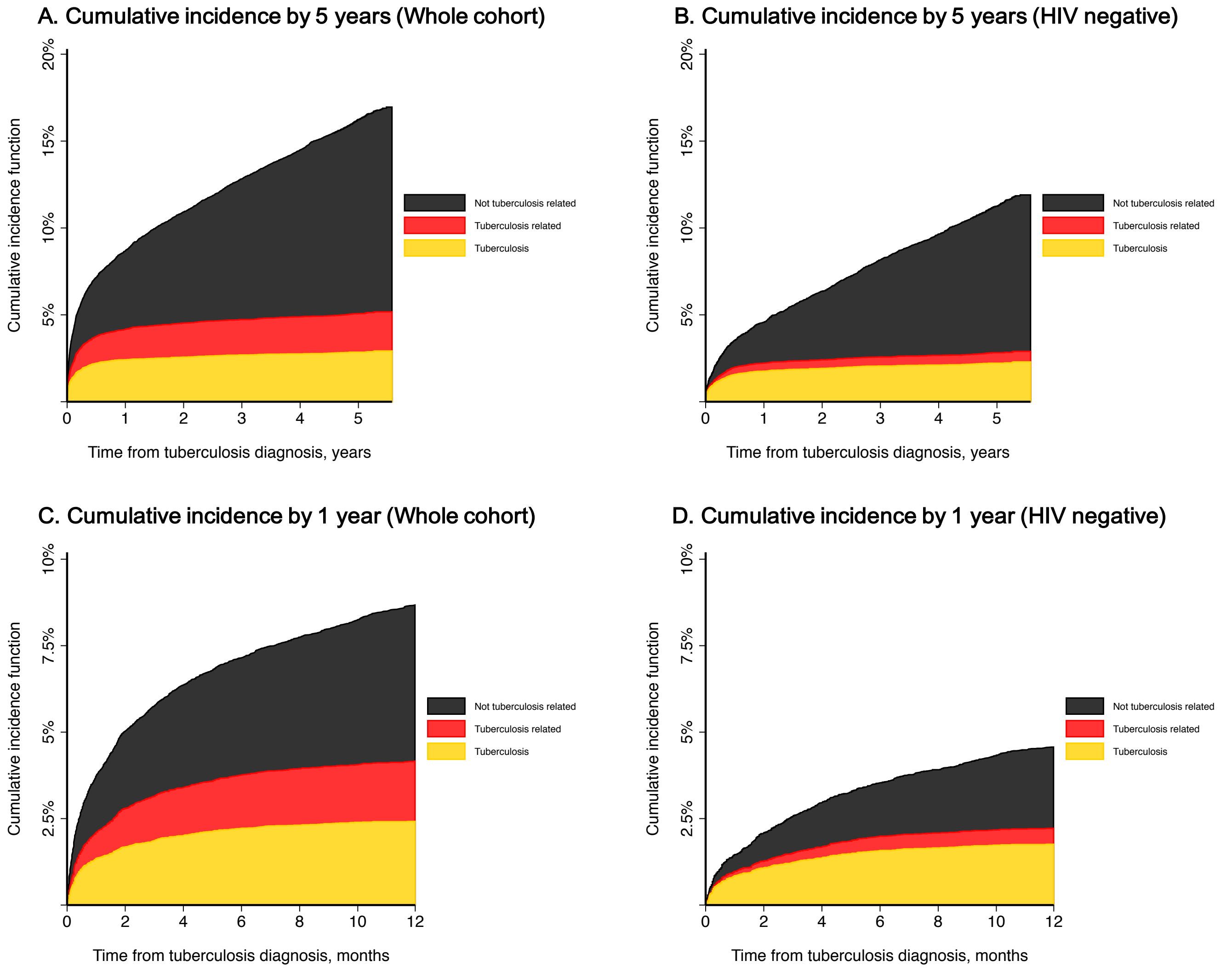

Tuberculosis refers to tuberculosis codes as the underlying cause of death. Tuberculosis related refers to tuberculosis codes in any other line in the death certificate.

eTable 21. The association between social vulnerability, health behaviours, and comorbidities and 5-year mortality related to any mention to tuberculosis in the death certificate of tuberculosis patients newly diagnosed in 2010 in São Paulo State, Brazil (sensitivity analysis for causes of death 2. Population: Whole and not immunocompromised cohorts).

		5-year cause-specific survival (n = 15342)	
**Exposures of interest**	**Values**	**Tuberculosis related (Whole cohort)**	**Tuberculosis related (Not immunocompromised)**
		**Adj. cHR (95% CI)[a], p-value**	**Adj. cHR (95% CI)[a], p-value**
**Social vulnerability**			
Inmate	Yes	0.68 (0.45-1.02), p=0.059	0.48 (0.19-1.18), p=0.110
Homelessness	Yes	1.51 (1.08-2.12), p=0.016	2.02 (1.25-3.27), p=0.004
**Health behaviours**			
Alcohol use	Yes	1.72 (1.24-2.39), p=0.001	1.89 (1.44-2.47), p<0.001
Drug use	Yes	1.15 (0.82-1.62), p=0.428	1.12 (0.69-1.82), p=0.638
**Comorbidities**			
Diabetes mellitus	Yes	0.85 (0.65-1.12), p=0.243	0.80 (0.53-1.21), p=0.298
Mental disorder	Yes	0.91 (0.59-1.40), p=0.667	1.00 (0.52-1.91), p=0.991
**Alcohol or drug use or homelessness**	Yes	1.64 (1.39-1.94), p<0.001	2.02 (1.56-2.60), p<0.001
**Alcohol or drug use or homelessness**	1 factor	1.55 (1.29-1.85)	1.94 (1.48-2.54)
	2 factors	1.93 (1.42-2.61)	2.19 (1.31-3.65)
	3 factors	2.99 (1.53-5.84)	5.57 (2.03-15.30)

[a] Fully adjusted models in 10 mulitple imputed datasets. Adj. cHR – adjusted cause-specific hazard ratios; CI – confidence interval.

eFigure 16. Fully adjusted cause-specific hazard ratios for 5-year tuberculosis related cause-specific mortality (sensitivity analysis for causes of death-2. Population: Whole and not immunocompromised cohorts)

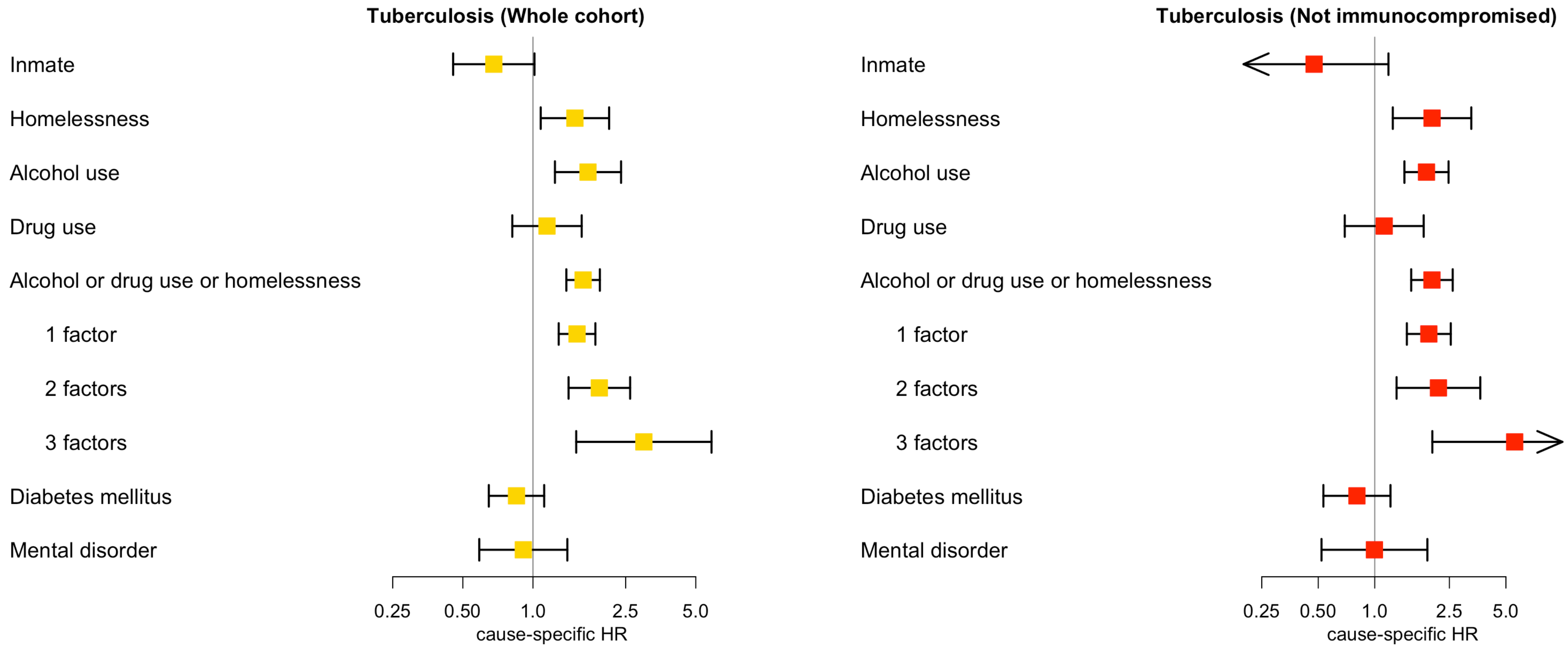

Adjusted cause-specific hazard ratios are shown as boxes and 95% confidence intervals as horizontal lines. The arrow represents the direction of the confidence interval value, truncated at the horizontal axes limit.
HR – hazard ratio

eTable 22. The association between social vulnerability, health behaviours, and comorbidities with 5-year mortality related to any mention to tuberculosis in the death certificate among those patients who survived the 1 in 2010 in São Paulo State, Brazil (sensitivity analysis for cause of death - 2).

		Cause-specific mortality among those patients alive after the 1-year (n = 14,011)
**Exposures of interest**	**Values**	**Tuberculosis related**
		**Adj. cHR (95% CI)[a], p-value**
**Social vulnerability**		
Inmate	Yes	0.77 (0.39-1.53), p=0.457
Homelessness	Yes	2.26 (1.14-4.48), p=0.020
**Health behaviours**		
Alcohol use	Yes	1.27 (0.66-2.45), p=0.481
Drug use	Yes	1.17 (0.58-2.35), p=0.662
**Comorbidities**		
Diabetes mellitus	Yes	0.79 (0.36-1.72), p=0.548
Mental disorder	Yes	0.44 (0.11-1.81), p=0.255
**Alcohol or drug use or homelessness**	Yes	1.83 (1.26-2.66), p=0.002
	1 factor	1.72 (1.14-2.6)
**Alcohol and drug use and homelessness**	2 factors	2.12 (1.08-4.15)
	3 factors	3.33 (0.8-13.8)

[a] Fully adjusted models in 10 mulitple imputed datasets. Adj. cHR – adjusted cause-specific hazard ratios; CI – confidence interval.

eFigure 17. Fully adjusted cause-specific hazard ratios for 5-year tuberculosis related cause-specific mortality among those alive after the 1-year (sensitivity analysis for causes of death-2)

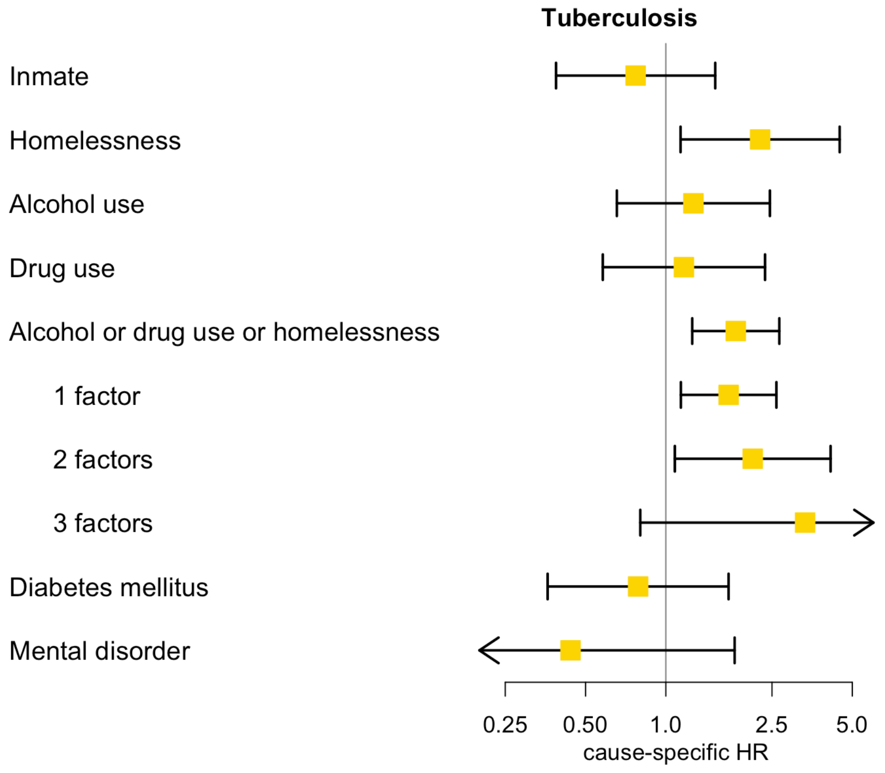

Adjusted cause-specific hazard ratios are shown as boxes and 95% confidence intervals as horizontal lines. The arrow represents the direction of the confidence interval value, truncated at the horizontal axes limit. HR – hazard ratio

REFERENCES

1. Galesi VM. [Data on tuberculosis in the state of São Paulo, Brazil]. *Rev Saude Publica* 2007; **41 Suppl 1**: 121.
2. Ranzani OT, Carvalho CR, Waldman EA, Rodrigues LC. The impact of being homeless on the unsuccessful outcome of treatment of pulmonary TB in Sao Paulo State, Brazil. *BMC Med* 2016; **14**: 41.
3. Magalhaes da Silva T, Sandhya Rani MR, de Oliveira Costa GN, et al. The correlation between ancestry and color in two cities of Northeast Brazil with contrasting ethnic compositions. *Eur J Hum Genet* 2015; **23**(7): 984-9.
4. Ranzani OT, Rodrigues LC, Waldman EA, Carvalho CRR. Estimating the impact of tuberculosis anatomical classification on treatment outcomes: A patient and surveillance perspective analysis. *PLoS One* 2017; **12**(11): e0187585.
5. World Health Organization-WHO. Definitions and reporting framework for tuberculosis - 2013 revision (updated December 2014). *WHO Publications* 2013: 1-47.
6. Sayers A, Ben-Shlomo Y, Blom AW, Steele F. Probabilistic record linkage. *Int J Epidemiol* 2016; **45**(3): 954-64.
7. Camargo KR, Jr., Coeli CM. [Reclink: an application for database linkage implementing the probabilistic record linkage method]. *Cad Saude Publica* 2000; **16**(2): 439-47.
8. Torrens A, Bartholomay P, Silva S, Khogali M, Verdonck K, Bissell K. HIV testing, antiretroviral therapy, and treatment outcomes in new cases of tuberculosis in Brazil, 2011. *Rev Panam Salud Publica* 2016; **39**(1): 26-31.
9. Rocha MS, Oliveira GPd, Aguiar FP, Saraceni V, Pinheiro RS. Do que morrem os pacientes com tuberculose: causas múltiplas de morte de uma coorte de casos notificados e uma proposta de investigação de causas presumíveis. *Cadernos de Saúde Pública* 2015; **31**: 709-21.
10. Lonnroth K, Jaramillo E, Williams BG, Dye C, Raviglione M. Drivers of tuberculosis epidemics: the role of risk factors and social determinants. *Soc Sci Med* 2009; **68**(12): 2240-6.
11. Hargreaves JR, Boccia D, Evans CA, Adato M, Petticrew M, Porter JD. The social determinants of tuberculosis: from evidence to action. *Am J Public Health* 2011; **101**(4): 654-62.
12. Lonnroth K, Castro KG, Chakaya JM, et al. Tuberculosis control and elimination 2010-50: cure, care, and social development. *Lancet* 2010; **375**(9728): 1814-29.
13. de Groot N, Bonsel GJ, Birnie E, Valentine NB. Towards a universal concept of vulnerability: Broadening the evidence from the elderly to perinatal health using a Delphi approach. *PLoS One* 2019; **14**(2): e0212633.
14. Shi L, Stevens GD, Lebrun LA, Faed P, Tsai J. Enhancing the measurement of health disparities for vulnerable populations. *J Public Health Manag Pract* 2008; **14 Suppl**: S45-52.
15. Story A, Murad S, Roberts W, Verheyen M, Hayward AC, London Tuberculosis Nurses N. Tuberculosis in London: the importance of homelessness, problem drug use and prison. *Thorax* 2007; **62**(8): 667-71.
16. Pareek M, Watson JP, Ormerod LP, et al. Screening of immigrants in the UK for imported latent tuberculosis: a multicentre cohort study and cost-effectiveness analysis. *Lancet Infect Dis* 2011; **11**(6): 435-44.
17. French CE, Kruijshaar ME, Jones JA, Abubakar I. The influence of socio-economic deprivation on tuberculosis treatment delays in England, 2000-2005. *Epidemiol Infect* 2009; **137**(4): 591-6.

18. Boccia D, Hargreaves J, Lönnroth K, et al. Cash transfer and microfinance interventions for tuberculosis control: review of the impact evidence and policy implications. *Int J Tuberc Lung Dis* 2011; **15 Suppl 2**: S37-49.
19. Tanimura T, Jaramillo E, Weil D, Raviglione M, Lonnroth K. Financial burden for tuberculosis patients in low- and middle-income countries: a systematic review. *Eur Respir J* 2014; **43**(6): 1763-75.
20. Munro SA, Lewin SA, Smith HJ, Engel ME, Fretheim A, Volmink J. Patient adherence to tuberculosis treatment: a systematic review of qualitative research. *PLoS Med* 2007; **4**(7): e238.
21. Waitt CJ, Squire SB. A systematic review of risk factors for death in adults during and after tuberculosis treatment. *Int J Tuberc Lung Dis* 2011; **15**(7): 871-85.
22. Dara M, Acosta CD, Melchers NV, et al. Tuberculosis control in prisons: current situation and research gaps. *Int J Infect Dis* 2015; **32**: 111-7.
23. World Health Organization-WHO. Global tuberculosis report 2018. Geneva; 2018.
24. Baker MA, Harries AD, Jeon CY, et al. The impact of diabetes on tuberculosis treatment outcomes: a systematic review. *BMC Med* 2011; **9**: 81.
25. Lederer DJ, Bell SC, Branson RD, et al. Control of Confounding and Reporting of Results in Causal Inference Studies. Guidance for Authors from Editors of Respiratory, Sleep, and Critical Care Journals. *Ann Am Thorac Soc* 2019; **16**(1): 22-8.
26. Rothman KJ, Greenland S, Lash TL. Modern epidemiology. 3rd ed. Philadelphia, Pa. ; London: Lippincott Williams & Wilkins; 2008.
27. Ranzani OT, Rodrigues LC, Waldman EA, Prina E, Carvalho CRR. Who are the patients with tuberculosis who are diagnosed in emergency facilities? An analysis of treatment outcomes in the state of Sao Paulo, Brazil. *J Bras Pneumol* 2018; **44**(2): 125-33.
28. Nogueira BMF, Rolla VC, Akrami KM, Kiene SM. Factors associated with tuberculosis treatment delay in patients co-infected with HIV in a high prevalence area in Brazil. *PLoS One* 2018; **13**(4): e0195409.
29. Reis-Santos B, Pellacani-Posses I, Macedo LR, Golub JE, Riley LW, Maciel EL. Directly observed therapy of tuberculosis in Brazil: associated determinants and impact on treatment outcome. *Int J Tuberc Lung Dis* 2015; **19**(10): 1188-93.
30. Sterne JA, White IR, Carlin JB, et al. Multiple imputation for missing data in epidemiological and clinical research: potential and pitfalls. *BMJ* 2009; **338**: b2393.
31. Bartlett JW, Seaman SR, White IR, Carpenter JR, Alzheimer's Disease Neuroimaging I. Multiple imputation of covariates by fully conditional specification: Accommodating the substantive model. *Stat Methods Med Res* 2015; **24**(4): 462-87.
32. Rubin DB. Multiple imputation for nonresponse in surveys. New York ;: Wiley; 1987.
33. Lambert PC, Royston P. Further development of flexible parametric models for survival analysis. *Stata Journal* 2009; **9**(2): 265-90.
34. Royston P, Parmar MK. Flexible parametric proportional-hazards and proportional-odds models for censored survival data, with application to prognostic modelling and estimation of treatment effects. *Stat Med* 2002; **21**(15): 2175-97.
